# Ongoing replication stress tolerance and clonal T cell responses distinguish liver and lung recurrence and outcomes in pancreatic cancer

**DOI:** 10.1038/s43018-024-00881-3

**Published:** 2025-01-09

**Authors:** Jason M. Link, Jennifer R. Eng, Carl Pelz, Kevin MacPherson-Hawthorne, Patrick J. Worth, Shamaline Sivagnanam, Dove J. Keith, Sydney Owen, Ellen M. Langer, Alison Grossblatt-Wait, Gustavo Salgado-Garza, Allison L. Creason, Sara Protzek, Julian Egger, Hannah Holly, Michael B. Heskett, Koei Chin, Nell Kirchberger, Konjit Betre, Elmar Bucher, David Kilburn, Zhi Hu, Michael W. Munks, Isabel A. English, Motoyuki Tsuda, Jeremy Goecks, Emek Demir, Andrew C. Adey, Adel Kardosh, Charles D. Lopez, Brett C. Sheppard, Alex Guimaraes, Brian Brinkerhoff, Terry K. Morgan, Gordon B. Mills, Lisa M. Coussens, Jonathan R. Brody, Rosalie C. Sears

**Affiliations:** 1 Department of Molecular and Medical Genetics, Oregon Health and Science University, Portland, OR USA; 2 Brenden-Colson Center for Pancreatic Care, Oregon Health and Science University, Portland, OR USA; 3 Knight Cancer Institute, Portland, OR USA; 4 Department of Surgery, Oregon Health and Science University, Portland, OR USA; 5 Department of Cell, Development and Cancer Biology, Oregon Health and Science University, Portland, OR USA; 6 Center for Early Detection Advanced Research, Oregon Health and Science University, Portland, OR USA; 7 Department of Biomedical Engineering, Oregon Health and Science University, Portland, OR USA; 8 Stanford Cancer Institute, Stanford University, Palo Alto, CA USA; 9 Department of Hematology and Oncology, Oregon Health and Science University, Portland, OR USA; 10 Department of Radiology, Oregon Health and Science University, Portland, OR USA; 11 Department of Pathology and Laboratory Medicine, Oregon Health and Science University, Portland, OR USA; 12 Department of Oncological Sciences, Oregon Health and Science University, Portland, OR USA

**Keywords:** Pancreatic cancer, Cancer genetics, Tumour biomarkers, Tumour immunology, Cancer

## Abstract

Patients with metastatic pancreatic ductal adenocarcinoma survive longer if disease spreads to the lung but not the liver. Here we generated overlapping, multi-omic datasets to identify molecular and cellular features that distinguish patients whose disease develops liver metastasis (liver cohort) from those whose disease develops lung metastasis without liver metastases (lung cohort). Lung cohort patients survived longer than liver cohort patients, despite sharing the same tumor subtype. We developed a primary organotropism (pORG) gene set enriched in liver cohort versus lung cohort primary tumors. We identified ongoing replication stress response pathways in high pORG/liver cohort tumors, whereas low pORG/lung cohort tumors had greater densities of lymphocytes and shared T cell clonal responses. Our study demonstrates that liver-avid pancreatic ductal adenocarcinoma is associated with tolerance to ongoing replication stress, limited tumor immunity and less-favorable outcomes, whereas low replication stress, lung-avid/liver-averse tumors are associated with active tumor immunity that may account for favorable outcomes.

## Abstract (web summary)

Sears and colleagues found that ongoing replication stress pathways and T cell clonal responses differentiate liver and lung recurrence and are associated with different clinical outcomes in the context of pancreatic ductal adenocarcinoma.

## Main

Patients with pancreatic ductal adenocarcinoma (PDAC) who present with metastatic disease (~50%) have a median survival of months. A subset of patients with PDAC (~10%) who develop primarily lung-restricted metastases survive significantly longer than patients with metastatic spread to other sites^1–3^; in some cases surviving >5 years with untreated, indolent lung metastases^4^ and may gain benefit from a metastatectomy^5^. In contrast, presentation with liver metastases or recurrent disease in the liver portends poor outcomes, partly a consequence of the liver’s immune suppressive tumor microenvironment (TME)^6–8^.

Many studies have categorized PDAC tumors into two to six subtypes based on gene expression in tumors^9–12^ and the surrounding TME^13,14^. Two consensus subtypes emerge from these studies^15^: the basal-like/quasi-mesenchymal/squamoid subtype and the classical/ductal/glandular subtype. Outcomes are poorer for patients with basal-like-subtype tumors. Basal-like tumors have been linked to gene expression signatures indicative of ongoing replication stress (RS)^16^, defined by stalled replication forks caused by premature entry into S phase, transcription/replication collisions or aberrant DNA damage checkpoints^17^. Failure to resolve RS leads to replication fork collapse, DNA damage, interferon (IFN) signaling, cell cycle arrest and, ultimately, senescence or cell death. Although aberrantly proliferating cancer cells are unavoidably plagued by RS, some malignant cells evolve response mechanisms to tolerate it, and their ability to survive the pro-mutagenic consequences of ongoing RS is likely key to their aggressive biology.

Ineffective PDAC tumor immunity and poor responses to immune checkpoint inhibitors (ICIs) contribute to aggressive, treatment-resistant PDAC^18,19^; however, exceptional cases exist, demonstrating that effective tumor immunity does occur naturally^20,21^. Future success with ICIs and other modulators of tumor immunity will likely require a better understanding of how rare cases of natural tumor immunity can control PDAC. In this study, we generated and interrogated large, overlapping datasets with genomic, transcriptomic and T cell receptor (TCR) blood and tumor sequencing of patient samples to evaluate tumor and immune differences between primary PDAC with liver versus lung metastatic organotropism. We report on both tumor-intrinsic and extrinsic features that distinguish liver-avid versus lung-avid, liver-averse PDAC independent from the known PDAC subtypes.

## Results

### Better outcomes in lung-avid/liver-averse metastatic PDAC

**Fig. 1:**
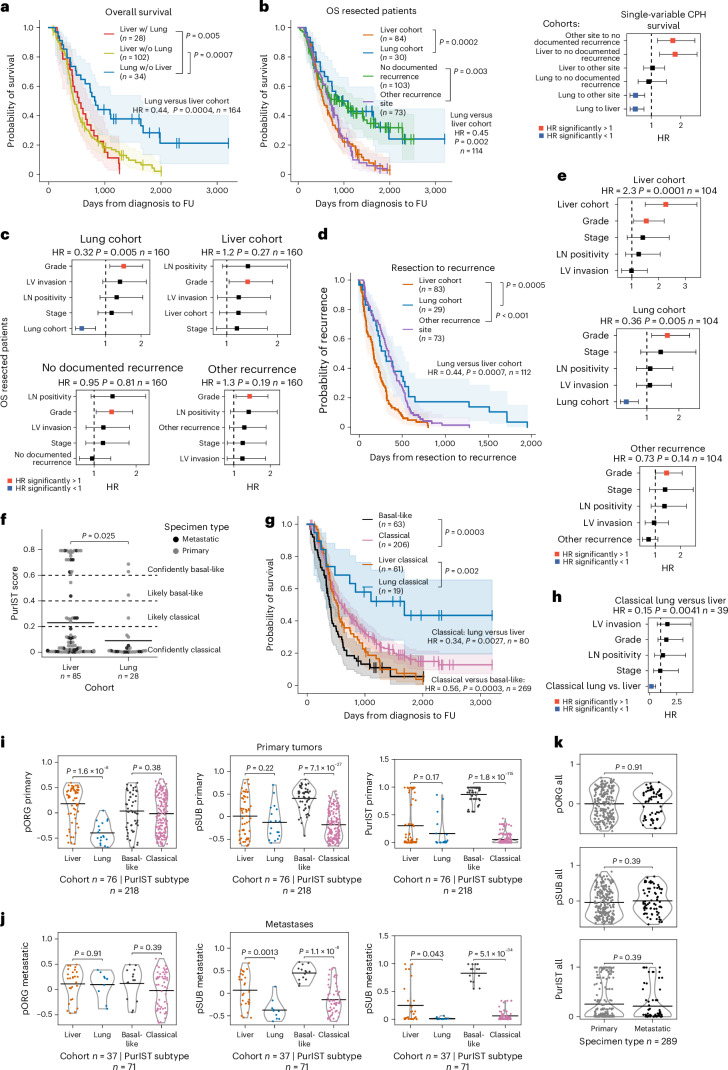
Survival outcomes and the primary organotropism gene set distinguish liver or lung recurrence independent of subtype. **a**, Kaplan–Meier (K–M) estimates of OS of all patients with documented liver (*n* = 102 patients (pts.)) and/or lung recurrence (*n* = 28 and 34 pts.), *P* = 0.0005 and *P* = 0.0007. **b**, OS of patients treated by resection stratified by metastatic cohort; documented liver metastases (*n* = 84 pts.) or lung metastases without liver metastases (*n* = 30 pts.; *P* = 0.0002), recurrent disease at nonliver/lung (other) sites (*n* = 73 pts.) or no documented recurrence (*n* = 103 pts.; *P* = 0.003); K–M estimates (left), CPH single-variable modeling (right). **c**, CPH multivariable modeling of OS for patients treated by resection stratified by metastatic cohort; lung metastases (*P* = 0.005), liver metastases (*P* = 0.27), no documented recurrence (*P* = 0.81) and recurrent disease at nonliver/lung (other) sites (*P* = 0.19) combined with clinical covariates significant in single-variable modeling (*n* = 160 pts. with clinical covariate data). **d**, K–M estimates of days between resection and recurrence for metastatic cohorts; liver metastases (*n* = 83 pts.), recurrent disease at nonliver/lung (other) sites (*n* = 73; *P* ≤ 0.0001), or lung metastases (*n* = 29 pts.; *P* = 0.0005). **e**, CPH multivariable modeling of days between resection and recurrence, stratified by metastatic cohort; liver metastases (*P* = 0.0001), lung metastases (*P* = 0.005) and recurrent disease at nonliver/lung (other) sites (*P* = 0.14) combined with clinical covariates (*n* = 104 pts. with clinical covariate data). **f**, PurIST subtyping scores for primary and metastatic tumor specimens from patients in the liver (*n* = 85 pts.) and lung (*n* = 28 pts.; *P* = 0.025) cohorts. Black bars represent means. *P* value from two-tailed *t*-test. **g**, K–M estimates of OS for patients categorized by PurIST subtype; basal-like (*n* = 63 pts.) or classical (*n* = 206 pts.; *P* = 0.0003) and liver/lung cohorts; liver classical (*n* = 61 pts.) or lung classical (*n* = 19 pts.; *P* = 0.002). **h**, CPH multivariable modeling of OS for classical subtype lung cohort versus classical subtype liver cohort patients (*n* = 39 pts.; *P* = 0.0041) combined with clinical covariates. **i**, GSVA scores for the pORG (left; liver or lung (*n* = 76, *P* = 1.6 × 10^−8^), basal-like or classical (*n* = 218, *P* = 0.38)) and pSUB gene sets (center; liver or lung (*n* = 76, *P* = 0.22), basal-like or classical (*n* = 218, *P* = 7.1 × 10^−27^)) and PurIST scores (right; liver or lung (*n* = 76, *P* = 0.17), basal-like or classical (*n* = 218, *P* = 1.8 × 10^−115^)) calculated from primary tumors. **j**, GSVA scores for the pORG (left; liver or lung (*n* = 37, *P* = 0.91), basal-like or classical (*n* = 71, *P* = 0.39)) and pSUB gene sets (center; liver or lung (*n* = 37, *P* = 0.0013), basal-like or classical (*n* = 71, *P* = 1.1 × 10^−8^)) and PurIST scores (right; liver or lung (*n* = 37, *P* = 0.043), basal-like or classical (*n* = 71, *P* = 5.1 × 10^−34^)) calculated from metastatic tumors. **k**, GSVA scores for primaries versus metastases for pORG (top (*n* = 289, *P* = 0.91)), pSUB (middle (*n* = 289, *P* = 0.39)) and PurIST scores (bottom (*n* = 289, *P* = 0.39)). Patients who died <30 days after resection were omitted (**a**–**e**,**g**,**h**). *P* values between groups indicated with brackets determined by log-rank test, shaded regions represent 95% confidence intervals (CIs), and HR, *P* value and *n* are from CPH single-variable modeling (**a**,**b**,**d**,**g**). HR and associated *P* value for recurrence site variable was determined by CPH modeling; squares mark the HR estimates, and the horizontal bars represent the 95% CI (**b**,**c**,**e**–**h**). Patients with complete information on covariates were included in CPH multivariable analysis. Black bars represent means; *P* values were derived from one-way analysis of variance (ANOVA) tests and corrected with the Benjamini–Hochberg method and *n* indicates number of tumors (**i**–**k**). FU, follow-up; LN, lymph node; LV, lymph/vascular. Source data

From a de-identified dataset of patients treated for PDAC at our institution with a complete set of disease-relevant computed tomography (CT) scans, we identified 35 patients who developed lung metastases but never developed evidence of liver metastases (hereafter referred to as the ‘lung cohort’); within this cohort, the shortest follow-up for patients alive at the time of data freeze was 760 days after resection and 984 days after diagnosis. We identified an additional 130 patients who developed liver metastases (referred to as the ‘liver cohort’), of which 28 also developed lung metastases. Consistent with previous reports^1–4^, we observed that lung cohort patients in our dataset fare significantly better by median overall survival (OS) than patients who developed liver metastases, regardless of whether they also developed lung metastases (819 (lung without liver) days versus 450 (liver without lung) or 537 (liver with lung) days; Fig. 1a). Median survival was also significantly longer for patients in the lung versus liver cohorts when limiting our analysis to patients treated by surgical resection (876 days versus 549 days, respectively; Fig. 1b). Patients with disease recurrence in sites other than liver or lung fared similarly to patients in the liver cohort (median survival, 693 days) and patients with no documented recurrence survived longer (median survival, 869 days; Fig. 1b).

**Extended Data Fig. 1:**
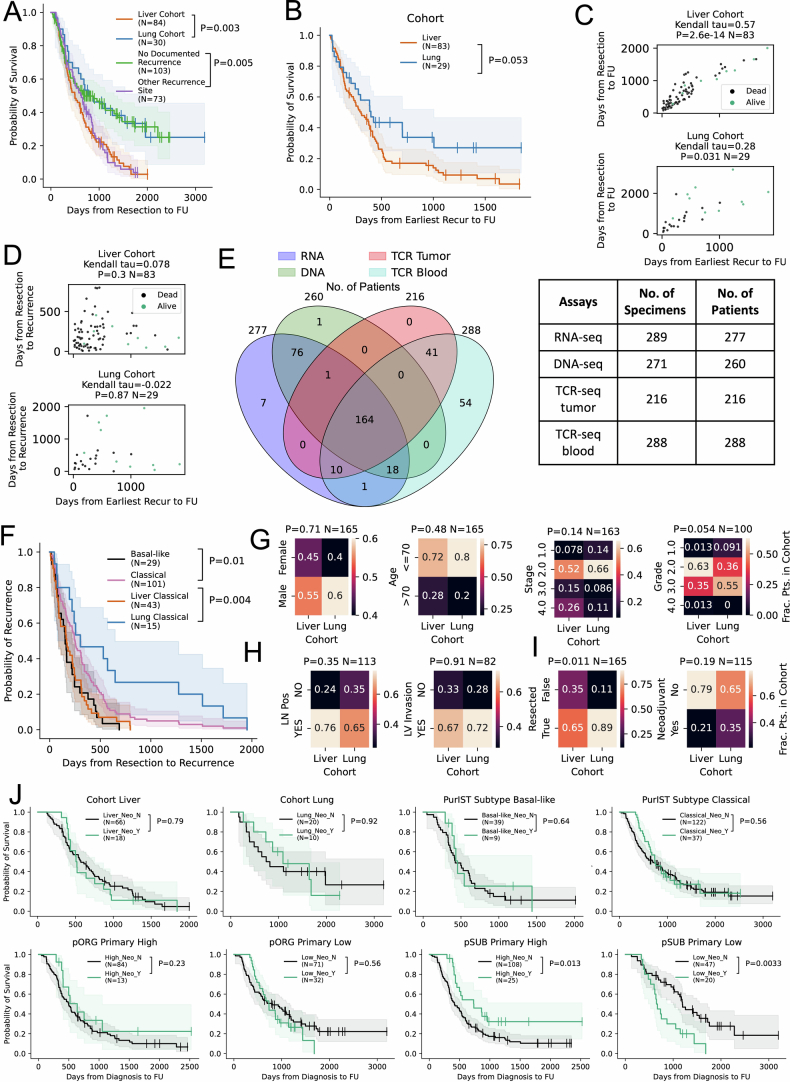
Survival and clinical characteristics of metastatic cohort and subtype. **A)** Kaplan–Meier (K–M) estimates of days from resection to follow-up for resected patients with known liver (N = 84 patients) and/or lung metastases (N = 30 patients, P = 0.003), other recurrence site (neither liver nor lung, N = 73 patients) or no documented recurrence (N = 103 patients, P = 0.005). **B)** K–M estimates of survival after liver (N = 83) or lung (N = 29, P = 0.053) recurrence. **C)** Kendall tau correlation (for censored data) between survival after liver (N = 83, P = 2.6e-14) or lung (N = 29, P = 0.031) recurrence and survival after resection. **D)** Kendall tau correlation between time to recurrence after resection and survival after liver (N = 83, P = 0.3) or lung (N = 29, P = 0.87) recurrence. **E)** Venn diagram of patient overlap (left, RNA-seq (N = 277), DNA-seq (N = 260], TCR-seq tumor (N = 216], TCR-seq blood (N = 288], and table of number of specimens (right, RNA-seq [N = 289], DNA-seq [N = 271], TCR-seq tumor [N = 216], TCR-seq blood [N = 288] with the indicated analyses. **F)** K–M estimates of days between resection and recurrence for all basal-like (N = 29) vs classical (N = 101, P = 0.01) patients, and all liver cohort (N = 43) vs lung cohort (N = 15, P = 0.004) classical patients. For the two patients with more than one specimen analyzed, the resected primary tumor was used for subtype assignment. **G-H)** Fraction of patients in liver or lung cohort with different clinical covariates, that is, male or female (N = 165, P = 0.71), age <= 70 or > 70 (N = 165, P = 0.48), stage (N = 163, P = 0.14), grade (N = 100, P = 0.054), LN positive (N = 113, P = 0.35), and LN invasion (N = 82, P = 0.91). **I)** Fraction of patients in liver or lung cohort receiving a resection (left, [N = 165, P = 0.011]) and the ratio of resected patients receiving neoadjuvant chemotherapy (right, [N = 115, P = 0.19]). **J)** K–M estimates of overall survival of resected patients stratified by neoadjuvant treatment (Neo) in the liver cohort (no neo [N = 66], neo [N = 18, P = 0.79 or lung cohort (no neo [N = 20], neo [N = 10, P = 0.92]), categorized by PurIST tumor subtype basal-like (no neo [N = 39], neo [N = 9, P = 0.64] or classical (no neo [N = 122], neo [N = 37, P = 0.56]), pORG primary high (no neo [N = 84], neo [N = 13, P = 0.23]) or pORG primary low (no neo [N = 71], neo [N = 32, P = 0.56] and pSUB primary high (no neo [N = 108], neo [N = 25, P = 0.013]) or pSUB primary low (no neo [N = 47], neo [N = 20, P = 0.00333]). **A-B, F, J)** Patients who died <30 days after resection were omitted. P values between groups indicated with brackets determined by log-rank test and shaded regions represent 95% confidence intervals. N=number of patients. **C-D)** Statistic and P value from two-sided Kendall tau correlation. N=number of patients. **G-I)** P value from Chi-squared test. N=number of patients. Source data

We performed multivariable analysis to account for clinical covariates that significantly correlated with survival in our dataset as single variables, including lymph/vascular invasion, grade, stage and lymph node positivity. Assignment to the lung cohort independently predicted longer survival for patients treated by resection in multivariable analysis, but assignment to other cohorts was not independently predictive of survival (Fig. 1c). Compared to patients in the liver cohort, lung cohort patients survived longer recurrence-free after resection (median 303 days versus 167 days, respectively; Fig. 1d) and survived longer overall after resection (median 784 versus 498 days, respectively; Extended Data Fig. 1a). By multivariable analysis, days from resection to recurrence for both liver and lung cohorts was significant independent of clinical covariates (Fig. 1e). Lung cohort patients generally survived longer after metastatic recurrence than the liver cohort (397 days versus 302 days, respectively, *P* = 0.053; Extended Data Fig. 1b) and survival after metastatic recurrence correlated with survival after resection (Extended Data Fig. 1c), but not with days from resection to recurrence (Extended Data Fig. 1d), suggesting biological differences in disease progression in the liver and lung cohorts between these two clinical time periods: before and after metastatic recurrence.

### Lung cohort survival advantage independent of tumor subtype

We generated gene expression data by performing RNA-seq on histologically confirmed tumor regions inclusive of integrated stroma from formalin-fixed paraffin-embedded (FFPE) primary (*n* = 218) and metastatic (*n* = 71) PDAC tumors (Extended Data Fig. 1e), and then used PurIST^12^ to assign consensus subtypes of PDAC (basal-like or classical) to each tumor. We found that tumors from lung cohort patients skewed significantly more classical than liver cohort tumors (Fig. 1f); and, as others have reported, patients with classical subtype tumors survived longer and had longer times to recurrence than patients with basal-like tumors (600 versus 394 days; Fig. 1g; and 250 versus 153 days; Extended Data Fig. 1f)^9,11^. When restricted to only patients with classical subtype tumors, the lung cohort survived longer and had later recurrence than the liver cohort (1,681 versus 520 days; Fig. 1g; and 303 versus 167 days; Extended Data Fig. 1f). These results highlight a subtype-independent survival benefit for patients in the lung cohort relative to the liver cohort, which is also independent of clinical covariates (hazard ratio (HR) = 0.15, *P* = 0.0041; Fig. 1h).

### Clinical comparisons reveal inflammation in lung cohort

**Extended Data Fig. 2:**
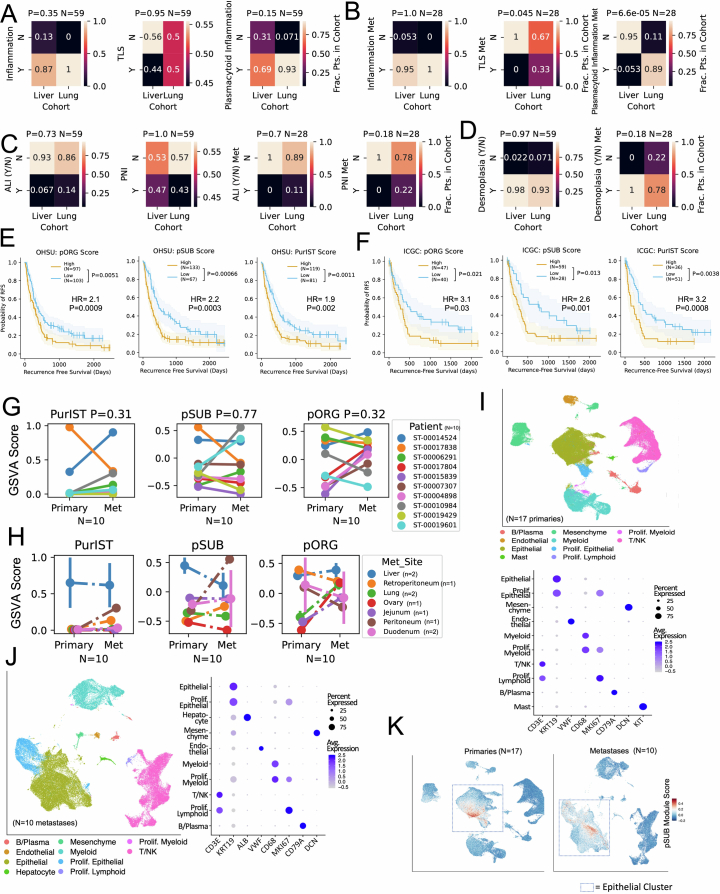
Organotropism and subtype association with histology, recurrence, and cell type. **A)** Fraction of patients in liver or lung cohort with different inflammatory features scored by a pathologist from hematoxylin and eosin (H&E) stained slides from the primary tumor that is, inflammation (N = 59, P = 0.35), TLS (N = 59, P = 0.95), and plasmacytoid inflammation (N = 59, P = 0.15). **B)** Fraction of patients in liver or lung cohort with different inflammatory features scored from metastatic tumor H&E slides that is, Metastases inflammation (N = 28, P = 1.0), TLS met (N = 28, P = 0.045), and plasmacytoid inflammation met (N = 28, P = 6.6e-5). **C)** Fraction of patients in liver or lung cohort with perineural invasion (PNI) from primary (N = 59, P = 2.0) or metastatic tumors (N = 28, P = 0.18) and angiolymphatic invasion (ALI) scored from H&E slides from the primary tumor (N = 59, P = 0.73) or metastatic tumor (N = 28, P = 0.7). **D)** Fraction of patients in liver or lung cohort with desmoplasia scored from H&E slides from the primary tumor (left, [N = 59, P = 0.97]) or metastatic tumor (right, [N = 28, P = 0.18]). **E)** Kaplan–Meier (K–M) estimates of recurrence-free survival (RFS) in OHSU patients split into high and low pORG (high,[N = 101], low [N = 107, P = 0.00062]), pSUB (high [N = 140], low [N = 68, P = 0.00013]) and PurIST (high [N = 126], low [N = 40, P = 0.021]) by cutoffs from Fig. 2d. **F)** K–M estimates of RFS in ICGC patients split into high and low pORG (high [N = 47], low [N = 82, P = 0.03]), pSUB (high [N = 59], low [N = 28, P = 0.001]) and PurIST (high [N = 26], low [N = 51, P = 0.0038]) by same cutoffs as OHSU patients. **G-H)** GSVA scores of matched primaries and mets from the same patient for PurIST (left, [N = 10 pts., P = 0.31]), pSUB (center, [N = 10 pts., P = 0.77]) and pORG (right, [N = 10 pts., P = 0.32]). **G)** colored by patient, P value from two-side Wilcoxon signed-rank test and **H)** grouped by met collection site showing mean GSVA (point) and 95% confidence intervals (error bars) (N = 10 pts. for all cohorts). **I-J)** Single-cell RNA-seq data from Werba G et al. (2023). **I)** UMAP of 17 primary PDAC tumors colored by cell types (top) and corresponding expression of cell type markers in each population (bottom). **J)** UMAP of 10 PDAC liver metastases colored by cell types (left) and corresponding cell type marker expression (right). **K)** pSUB module scores in primary tumors (left) and PDAC liver metastases (right). **A-D)** P value from chi-squared test. Plasmacytoid inflammation defined as the presence of plasma cells in a background of chronic inflammation (that is lymphocytes). Lymphoid aggregates/tertiary lymphoid structures (TLS) are specifically defined as clusters of lymphocytes forming a reactive germinal center in the tissue. PNI: Perineural invasion requires the carcinoma invades into the perineurial space around nerves. ALI: angiolymphatic invasion, defined as the presence of tumor cells within venous or lymphatic spaces. Desmoplasia is defined as dense fibrosis with elastin and collagen deposition around invading tumor cells. N=number of patients. **E-F)** P values between groups indicated with brackets determined by log-rank test and shaded regions represent 95% confidence intervals. N=number of patients. Source data

We did not observe significant differences in sex, age, stage at diagnosis, tumor grade, lymph-vascular invasion or lymph node positivity between patients in the lung and liver cohorts (Table 1 and Extended Data Fig. 1g,h). Patients in the lung cohort were more likely to be treated by resection than patients in the liver cohort (89% versus 65%, respectively; Extended Data Fig. 1i); however, the survival advantage in the lung cohort is still evident when only comparing patients treated by resection (Fig. 1b). A small fraction of patients in this dataset were treated with standard-of-care neoadjuvant chemotherapy in both cohorts (Extended Data Fig. 1i), but neoadjuvant treatment did not influence OS (Extended Data Fig. 1j). By histopathology, significantly more lung cohort tumors had chronic inflammation and plasmacytoid inflammation (Table 1). Inflammatory scores were not different between the two cohorts when comparing only resected primary tumors (Extended Data Fig. 2a). More lung cohort metastases had tertiary lymphoid structures/lymphoid aggregates (TLSs/LAs) (Extended Data Fig. 2b). Perineural invasion, angiolymphatic invasion and desmoplasia were not significantly different in liver versus lung cohort primaries or metastases (Extended Data Fig. 2c,d).

**Table 1:** Patient demographics, disease characteristics and tumor specimen histology parameters for all patients in study and subsets categorized into liver and lung cohorts or high and low pORG score in primary tumors

		Total	Liver cohort	Lung cohort	High pORG (primary tumors)	Low pORG (primary tumors)
Clinical characteristics		*n* = 422	*n* = 130	*n* = 35	*n* = 103	*n* = 104
Sex	Female	193 (46%)	59 (45%)	14 (40%)	50 (49%)	43 (41%)
	Male	229 (54%)	71 (55%)	21 (60%)	53 (51%)	61 (59%)
Race	White	386 (91%)	119 (92%)	31 (88.5%)	95 (92%)	100 (96%)
	Asian	13 (3%)	5 (3%)	1 (3%)	2 (2%)	1 (1%)
	Unknown	23 (5%)	6 (5%)	3 (8.5%)	6 (6%)	3 (3%)
Primary tumor site	Pancreas (adenocarcinoma only)	413 (98%)	127 (98%)	35 (100%)	99 (96%)	103 (99%)
	Ampulla of Vater (pancreaticobiliary type only)	9 (2%)	3 (2%)	0 (0%)	4 (4%)	1 (1%)
Stage	Stage 0	2 (0.5%)	0 (0%)	0 (0%)	1 (1%)	0 (0%)
	Stage 1a	8 (1.9%)	1 (1%)	1 (3%)	3 (3%)	3 (3%)
	Stage 1b	33 (7.8%)	9 (7%)	4 (11%)	4 (4%)	8 (8%)
	Stage 2a	63 (14.9%)	15 (12%)	6 (17%)	**13 (13%)**	**25 (24%)**
					***P*** = **0.047**	
	Stage 2b	174 (41.2%)	51 (39%)	17 (49%)	66 (64%)	57 (55%)
	Stage 3	54 (12.8%)	19 (15%)	3 (9%)	9 (9%)	10 (10%)
	Stage 4	73 (17.3%)	33 (25%)	4 (11%)	**7 (7%)**	**0 (0%)**
					***P*** = **0.0068**	
	No data	15 (3.6%)	2 (2%)	0 (0%)	0 (0%)	1 (1%)
Grade	1 – Well differentiated	11 (2.6%)	1 (1%)	2 (6%)	2 (2%)	6 (6%)
	2 – Moderately differentiated	127 (30.1%)	49 (38%)	8 (23%)	45 (44%)	43 (41%)
	3 – Poorly differentiated	85 (20.1%)	27 (21%)	12 (34%)	32 (31%)	30 (29%)
	4 – Undifferentiated	2 (0.5%)	1 (1%)	0 (0%)	1 (1%)	1 (1%)
	Not determined	197 (46.7%)	52 (40%)	13 (37%)	23 (22%)	24 (23%)
Treated by resection		*n* = 298	***n*** = **84 (65%)**	***n*** = **31 (89%)**	*n* = 99 (96%)	*n* = 100 (96%)
			***P*** = **0.007**			
Resection details	Neoadjuvant treatment	70 (24%)	18 (21%)	11 (35%)	**14 (14%)**	**32 (32%)**
					***P*** = **0.004**	
	No residual tumor	240 (81%)	74 (88%)	28 (90%)	78 (79%)	76 (76%)
	Residual disease present	55 (19%)	10 (12%)	3 (10%)	21 (21%)	22 (22%)
	Angiolymphatic invasion	145 (49%)	43 (51%)	13 (42%)	52 (53%)	46 (46%)
	Tumor involved in regional lymph nodes	205 (69%)	63 (75%)	20 (65%)	76 (77%)	64 (64%)
Histology analysis performed		*n* = 239	*n* = 64	*n* = 23	*n* = 83	*n* = 85
Histology	Acute inflammation	126 (53%)	35 (55%)	9 (39%)	46 (55%)	35 (41%)
	Chronic inflammation	196 (82%)	**46 (72%)**	**22 (96%)**	72 (87%)	71 (84%)
			***P*** = **0.019**			
	Plasmacytoid inflammation	148 (62%)	**32 (50%)**	**21 (91%)**	61 (73%)	61 (72%)
			***P*** = **0.0004**			
	LAs/TLSs	78 (33%)	20 (31%)	10 (43%)	33 (40%)	38 (45%)
	Perineural invasion	75 (31%)	21 (33%)	8 (35%)	35 (42%)	34 (40%)
	Desmoplasia	232 (97%)	63 (98%)	20 (87%)	83 (100%)	81 (95%)

Percentages for resection details are only from primary tumor resections and percentages for histology are only from tumors with histology analyzed. Comparisons significantly different between liver and lung cohort (all tumors) or high and low pORG primary tumors are shown in bold (*P* value below cells compared is from two-tailed Fisher’s exact test). Histology, review of H&E-stained sections by two board-certified pathologists blinded to study cohorts. Acute inflammation is defined as increased numbers of neutrophils compared to normal controls. Chronic inflammation is defined as increased numbers of lymphocytes. Plasmacytoid inflammation is defined as the presence of plasma cells in a background of chronic inflammation. LAs/TLSs are specifically defined as clusters of lymphocytes forming a reactive germinal center in the tissue. Perineural invasion requires the carcinoma invades into the perineurial space around nerves. Angiolymphatic invasion is defined as the presence of tumor cells within venous or lymphatic spaces. Desmoplasia is defined as dense fibrosis with elastin and collagen deposition around invading tumor cells.

### Subtype-independent organotropism gene set predicts survival

We sought to identify gene expression in primary tumors associated with liver-avidity versus lung avidity/liver aversion without being influenced by the higher percentage of basal-like tumors in the liver cohort (Fig. 1f). We ran a two-factor analysis with DESeq2 (ref. ^22^) to identify differentially expressed (DE) genes in primary tumors from the liver cohort versus lung cohort (organotropism) and from the basal-like versus the classical subtype. To focus on the biology of metastatic organotropism independent from subtype^12^, we excluded the top DE genes for subtype from the DE genes for organotropism to generate a primary organotropism gene set termed pORG (55 upregulated genes). We also applied this process to the DE genes from basal-like versus classical, subtracting the top DE organotropism genes to generate a gene set termed pSUB (primary tumor subtype; 51 upregulated genes).

We used Gene Set Variation Analysis (GSVA)^23^ to generate activity scores of our primary tumor samples for both the pORG and pSUB gene sets. As expected, pORG scores for liver cohort primary tumors were significantly higher than those from the lung cohort, but pORG score did not significantly separate primary basal-like tumors from classical tumors (Fig. 1i, left). Conversely, pSUB scores were significantly higher for basal-like than classical tumors but not different between liver and lung cohort primary tumors, similar to PurIST (Fig. 1i, center and right). The pORG scores for metastatic samples did not distinguish liver cohort from lung cohort metastases (Fig. 1j, left). The pSUB score distinguished basal-like from classical metastases (Fig. 1j, center) and also distinguished metastases from the liver and lung cohorts, similar to PurIST (Fig. 1j, right). GSVA scores for all specimens showed a similar spread in scores between all primary and all metastatic tumors for pORG, pSUB or PurIST (Fig. 1k).

**Fig. 2:**
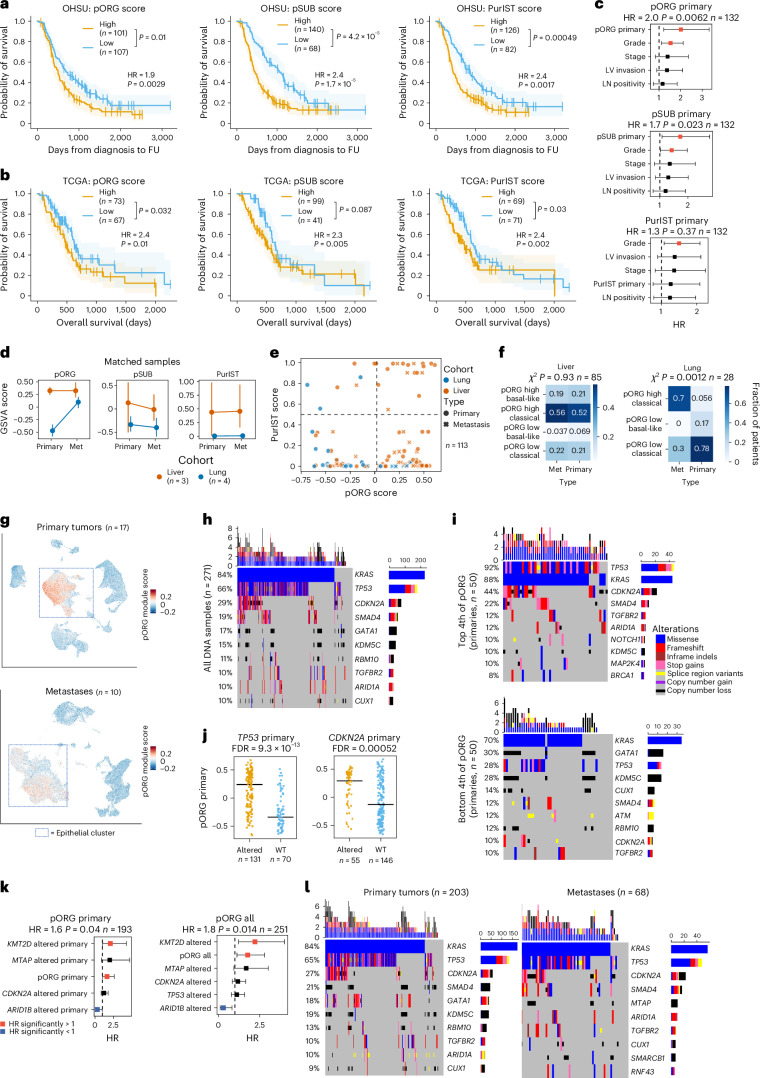
pORG predicts survival independently of clinical and genomic features. **a**, K–M estimate of OS for patients with primary tumors having high or low pORG (left; high (*n* = 101 pts.), low (*n* = 107 pts.; *P* = 0.01)), pSUB (middle; high (*n* = 140 pts.), low (*n* = 68 pts.; *P* = 4.2× 10^−5^)) or PurIST scores (right; high (*n* = 126 pts.), low (*n* = 82 pts.; *P* = 0.00049)) from the OHSU dataset. High/low risk was determined by receiver operating characteristic curve (ROC) and maximum Youden’s index. **b**, K–M estimate of OS in TCGA pORG (left; high (*n* = 73 pts.), low (*n* = 67 pts.; *P* = 0.032), pSUB (middle; high (*n* = 99 pts.), low (*n* = 41 pts.; *P* = 0.087) or PurIST (right; high (*n* = 69 pts.), low (*n* = 71 pts.; *P* = 0.03)) patients with PDAC. High/low score is defined using cutoff from OHSU dataset. **c**, CPH multivariable modeling of OS versus primary GSVA score for pORG (top; *P* = 0.0062), pSUB (middle; *P* = 0.023) and PurIST (bottom; *P* = 0.37) with clinical covariates (*n* = 132 pts.). **d**, pORG, pSUB and PurIST scores of primaries and metastases (Met) from the same patient, grouped by clinically defined liver cohort (documented liver recurrence, *n* = 3 pairs) or lung cohort (documented lung recurrence without liver recurrence, *n* = 4 pairs) showing cohort mean GSVA (point) and 95% CI (error bars). **e**, pORG and PurIST scores for primaries (circles) and metastases (x) in liver and lung cohorts (*n* = 113 pts.). **f**, Fraction of primaries or metastases in each quadrant of the graph in **e**; liver (*n* = 85 pts., *P* = 0.93) or lung (*n* = 28 pts., *P* = 0.0012). *P* values from two-way chi-squared test between primary and metastatic specimens. **g**, UMAP of Werba et al.^25^ scRNA-seq, shaded by per-cell scores for pORG in PDAC primaries (top; *n* = 17 pts.) and PDAC liver metastases (bottom; *n* = 10 pts.). **h**,**i**, Oncoprints of the top ten altered genes and alteration types (*n* = 271 tumors) in the DNA dataset (**h**) and top (above, *n* = 50 pts.) and bottom quartile (below, *n* = 50 pts.) (**i**) by pORG primary GSVA score. **j**, pORG primary GSVA score versus *TP53* (left; altered (*n* = 131 pts.), WT (*n* = 70 pts.), FDR = 9.3 × 10^−13^) or *CDKN2A* (right; altered (*n* = 55 pts.), WT (*n* = 146 pts.), FDR = 0.00052) gene alteration. *P* value from two-tailed *t*-test calculated for genes with ≥10 alterations in the dataset, corrected with the Benjamini–Hochberg method. **k**, CPH multivariable modeling of OS versus pORG GSVA score and genomic alterations prognostic in single-variable CPH modeling in primary tumors (left; *n* = 193 pts., *P* = 0.04) and all tumors (right; *n* = 251 pts., *P* = 0.014). **l**, Oncoprints of the top ten altered genes and their alteration types in primaries (left; *n* = 203 tumors) and metastases (right; *n* = 68 tumors). The log-rank test *P* values and *n* per group are indicated with brackets, shaded regions represent 95% CI, and CPH single-variable modeling HRs and associated *P* values are displayed on plots (**a**,**b**). Frequency is indicated at left, top bars indicate variant types by tumor, and right bars indicate variant types by gene (**h**,**i**,**l**). Alteration key (**i**). HR and associated *P* value for GSVA or PurIST score was determined by CPH modeling, squares represent HR estimates, and error bars represent 95% CIs (**c**,**k**). Patients who died within 30 days after resection are not shown (**a**,**c**,**k**). Source data

We found significant differences in OS between patients with tumors scoring high versus low for pORG, pSUB and PurIST (Fig. 2a), as well as significantly different recurrence-free survival (RFS; Extended Data Fig. 2e). Using the same high/low risk cutoffs for pORG, pSUB and PurIST scores determined in our dataset (Fig. 2a), pORG, pSUB and PurIST similarly predicted survival in two external datasets: OS in the pancreatic adenocarcinoma patient dataset (PAAD)^24^, reported by The Cancer Genome Atlas (TCGA) (cBioPortal) (Fig. 2b), and RFS in the Australian Pancreatic Cancer Genome Initiative (APGI)^9^, part of the International Cancer Genome Consortium (ICGC) study (Extended Data Fig. 2f). Low pORG primary tumors were more likely to be early stage and treated with neoadjuvant chemotherapy (Table 1); however, neoadjuvant treatment did not affect OS (Extended Data Fig. 1j). Multivariable analysis indicated that both pORG and pSUB predicted survival independently from other clinical covariates, but PurIST was influenced by grade (Fig. 2c).

Analysis of pORG, pSUB and PurIST scores in ten matched primary tumors and metastases (Extended Data Fig. 2g) revealed that lung metastases (*n* = 2 pairs) and metastases in the clinically defined lung cohort (*n* = 4 pairs) went from low in primaries to high in metastases, whereas liver cohort primaries and metastases stayed high (Fig. 2d and Extended Data Fig. 2h); consistent with pORG not distinguishing between liver and lung cohort metastatic samples (Fig. 1j). In unpaired primaries and metastases, the liver cohort had a similar fraction of low pORG samples in primaries and metastases, whereas lung cohort metastases show a shift to 70% high pORG (Fig. 2e,f). Although the metastatic TME may contribute to this shift in gene expression, analysis of a publicly available single-cell RNA-seq dataset^25^ demonstrated that both the pORG and pSUB gene sets are enriched in the epithelial cell populations from PDAC primaries and liver metastases (Fig. 2g and Extended Data Fig. 2i–k).

### *TP53* and *CDKN2A* alterations are enriched in high pORG tumors

**Extended Data Fig. 3:**
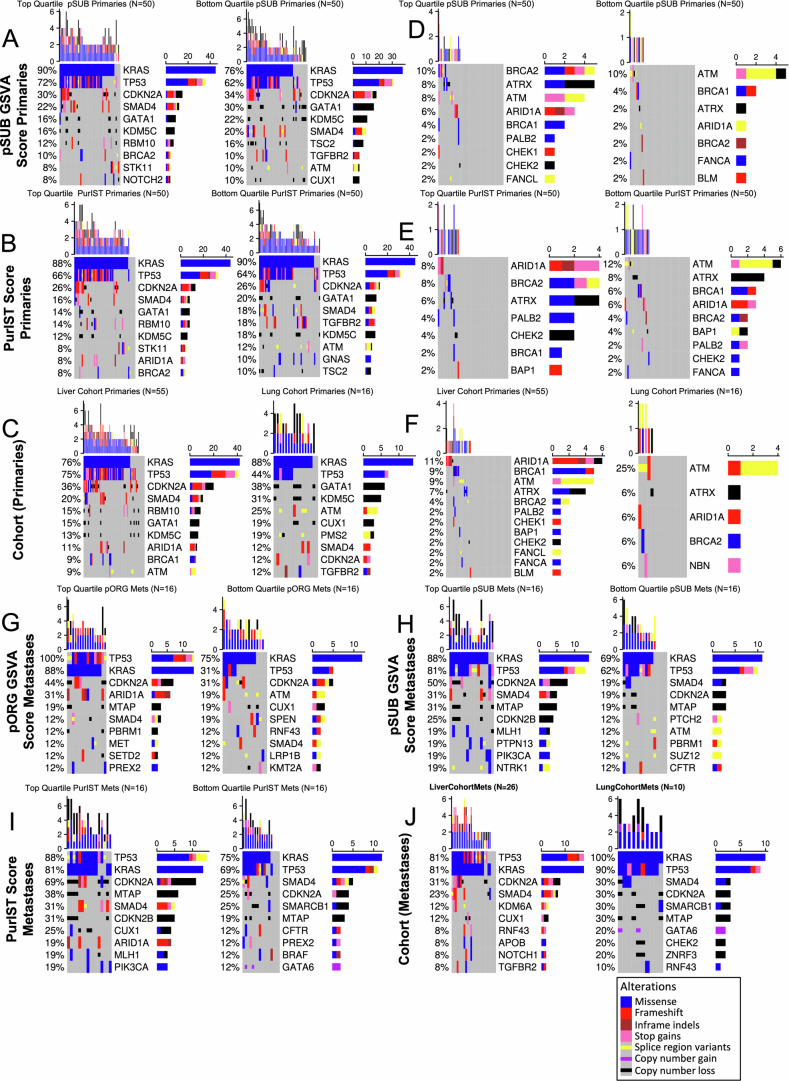
Genomic alterations associated with metastatic cohort, organotropism, and subtype. **A-C)** Oncoprints indicating the top ten most frequently altered genes and their alteration types in primary tumors in the top quartile (left) versus bottom quartile (right) by **A)** pSUB primary GSVA score (top quartile [N = 50], or bottom quartile [N = 50]) **B)** PurIST primary score (top quartile [N = 50], bottom quartile [N = 50]) and **C)** liver (left, [N = 55]) and lung (right, [N = 16]) cohort primary tumors. **D-F)** Oncoprints indicating the top ten most frequently altered DDR-relevant genes and their alteration types in primary tumors in the top quartile (left) versus bottom quartile (right) by **D)** pSUB primary GSVA score (top quartile [N = 50], bottom quartile [N = 50] **E)** PurIST primary score and **F)** liver (left, [N = 55]) and lung (right, [N = 16]) cohort primary tumors. **G-J)** Oncoprints indicating the top ten most frequently altered genes and their alteration types in metastatic tumors in the top quartile (left) versus bottom quartile (right) by **G)** pORG metastatic GSVA score (top quartile [N = 16], bottom quartile [N = 16]) **H)** pSUB metastatic GSVA score (top quartile [N = 16], bottom quartile [N = 16]) **I)** PurIST metastatic score (top quartile [N = 16], bottom quartile [N = 16]) and **J)** liver (left, [N = 26]) and lung (right, [N = 16]) cohort metastatic tumors. **A-J)** To the left of each panel is the gene alteration frequency in the cohort, the top bars indicate variant types by tumor, and right bars indicate variant types by gene. Variant type legend in lower right of figure. N=number of patients. Source data

**Extended Data Fig. 4:**
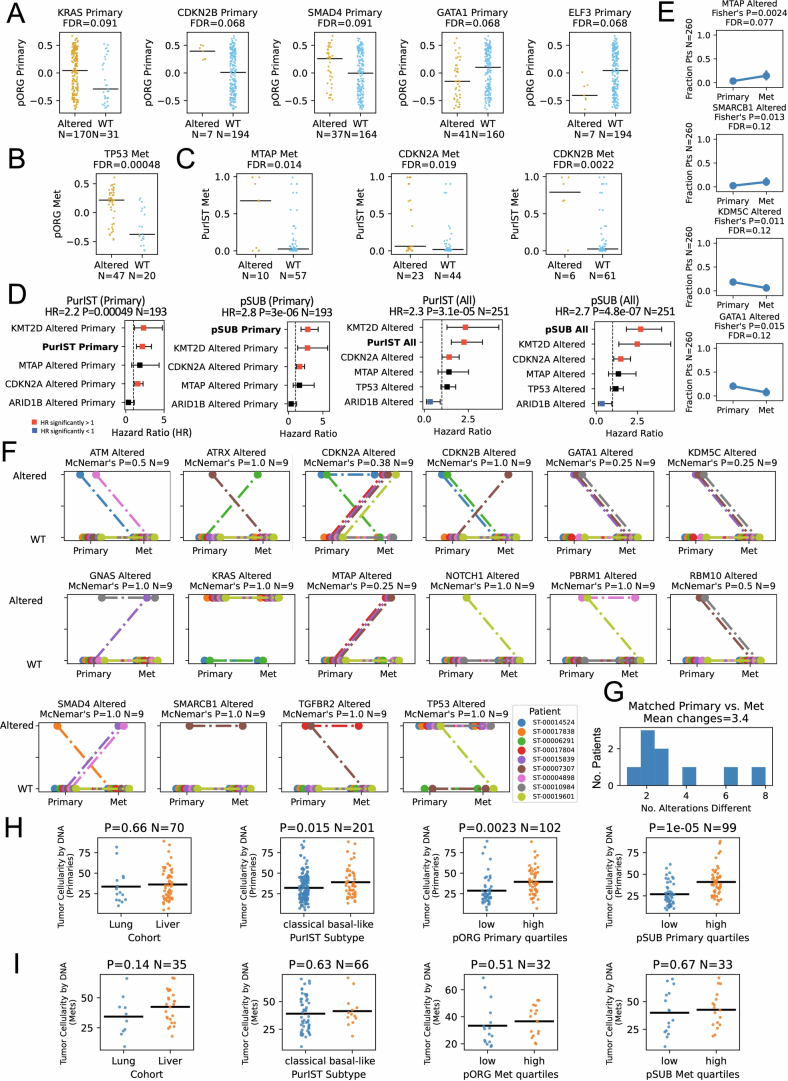
Gene alteration and tumor cellularity association with organotropism, subtype, distant recurrence, and survival. **A-C)** Gene alteration status (Altered or WT) versus GSVA score of **A)** pORG in primary tumors that is KRAS (altered [N = 170], WT [N = 31, FRD = 0.091]), CDKN2B (altered [N = 7], WT [N = 194, FDR = 0.068]), SMAD4 (altered [N = 37], WT [N = 164, FDR = 0.091]), GATA1 (altered [N = 41], WT [N = 160, FDR = 0.068]), and ELF3 (altered [N = 7], WT [N = 194, FDR = 0.068]). **B)** pORG in metastases that is TP53 (altered [N = 47], WT [N = 20, FDR = 0.00048]). **C)** PurIST in metastases that is MTAP (altered [N = 10], WT [N = 57, FDR = 0.014]), CDKN2A (altered [N = 23], WT [N = 44, FDR = 0.019]), and CDKN2B (altered [N = 6], WT [61, FDR = 0.0022]). **D)** Cox proportional hazard multi-variable modeling of overall survival versus PurIST (primary [N = 193, P = 0.00049] or all [N = 251, P = 3.1e-05]) or pSUB (primary [N = 193, P = 3e-06], or all [N = 251, P = 4.8e-07]) GSVA score combined with genomic alterations that were prognostic in single-variable CPH. HR and associated P value for variable in bold was determined by CPH modeling. Squares indicate hazard ratio estimates, and error bars show 95% confidence interval. Patients who died within 30 days after resection are not shown. **E)** Fraction of tumors altered for genes with significantly different alteration frequency between primaries and mets that is MTAP (N = 260, Fisher’s P = 0.0024, FDR = 0.077), SMARCB1 (N = 260, Fisher’s P = 0.013, FDR = 0.12), KDM5C (N = 2600, Fisher’s P = 0.011, FDR = 0.12) and GATA1 (N = 260, Fisher’s P = 0.015, FDR = 0.12). P value from Fisher’s exact test corrected with the Benjamini/Hochberg method. **F)** Alteration status of genes with at least one alteration in nine patients with matched primaries and mets that is ATM (N = 9, McNemar’s P = 0.5), ATRX (N = 9, McNemar’s P = 1.0), CDKN2A (N = 9, McNemar’s P = 0.38), CDKN2B (N = 9, McNemar’s P = 1.0), GATA1 (N = 9, McNemar’s P = 0.25), KDM5C (N = 9, McNemar’s P = 0.25), GNAS (N = 9, McNemar’s P = 2.0), KRAS (N = 9, McNemar’s P = 1.0), MTAP (N = 9, McNemar’s P = 0.25), NOTCH1 (N = 9, McNemar’s P = 1.0), PBRM1 (N = 9, McNemar’s P = 1.0), RBM10 (N = 9, McNemar’s P = 0.5), SMAD4 (N = 9, McNemar’s P = 1.0), SMARCB1 (N = 9, McNemar’s P = 1.0), TGFBR2 (N = 9, McNemar’s P = 1.0), and TP53 (N = 9, McNemar’s P = 1.0). P value from McNemar’s test. **G)** Histogram of number of alteration differences between matched primary and met from the same patient (N = 9 patients with DNA sequencing). **H)** The percentage of tumor cells in primary tumor samples analyzed by RNA-seq determined by mutant allele frequencies from the amplicon-based, high-throughput sequencing of 595 genes on the Tempus xT genomic alteration panel for the indicated comparisons that is lung or liver (N = 70, P = 0.66) classical or basal-like PurIST subtype (N = 201, P = 0.015), low or high pORG primary quartiles (N = 102, P = 0.0023), and low or high pSUB primary quartiles (N = 99. P = 1e-05). **I)**. The percentage of tumor cells in metastatic tumor samples analyzed by RNA-seq determined as in (**C**) for the indicated comparisons that is lung or liver (N = 35, P = 0.14) classical or basal-like PurIST subtype (N = 66, P = 0.63), low or high pORG met quartiles (N = 32, P = 0.51), and low or high pSUB met quartiles (N = 33. P = 0.67). **A-C)** For genes with >10 alterations in the dataset, P values obtained from two-tailed t-test and corrected with the Benjamini/Hochberg method. Black bars represent means. N=number of patients. **H-I)** P values from two-tailed t-test. Black bars represent means. N=number of patients. Source data

We used a tumor-relevant, 595 gene sequencing panel to analyze DNA alterations from the same specimens used for RNA sequencing (RNA-seq) (271 specimens with DNA data; Fig. 2h) and compared and ranked gene alterations between liver and lung cohorts, and high and low pORG, pSUB and PurIST quartiles (Fig. 2i and Extended Data Fig. 3a–j). *TP53* and *CDKN2A* altered primaries had significantly higher pORG GSVA scores (Fig. 2j), whereas *KRAS*, *CDKN2B* and *SMAD4* altered primaries trended higher and *GATA1* and *ELF3* altered primaries trended lower in pORG score (Extended Data Fig. 4a). In metastases, *TP53* altered tumors had higher pORG scores (Extended Data Fig. 4b); and *MTAP*, *CDKN2A* and *CDKN2B* altered tumors had higher PurIST scores (Extended Data Fig. 4c). We used Cox proportional hazards (CPH) multivariable modeling for OS against pORG score combined with gene alterations that were prognostic as single variables (*TP53*, *CDKN2A*, *KMT2D*, *MTAP* and *ARID1B*) and found that pORG score predicted shorter survival independent of genomic alterations in primaries and all samples (Fig. 2k), as did pSUB and PurIST scores (Extended Data Fig. 4d). We examined alteration differences between all primaries and all metastases in our dataset and in nonmatched samples we found *MTAP* and *SMARCB1* trended toward more alterations in metastases compared to primaries, whereas *KDM5C* and *GATA1* trended toward fewer alterations in metastases (Fig. 2l and Extended Data Fig. 4e). We found no consistent changes across nine paired primary tumors and metastases (Extended Data Fig. 4f). There was an average of 3.4 differences in genetic alterations between paired primaries and metastases from our gene panel, consistent with stochastic changes in matched samples (Extended Data Fig. 4g). DNA analysis indicated higher tumor cell content in basal-like versus classical primary tumors, consistent with another report^14^, and in high pORG and high pSUB primary tumors, but no significant difference between the liver and lung cohort primary tumors; or metastases in any of the groups (Extended Data Fig. 4h–i).

### Distinct pathways enriched by pORG and pSUB gene sets

**Fig. 3:**
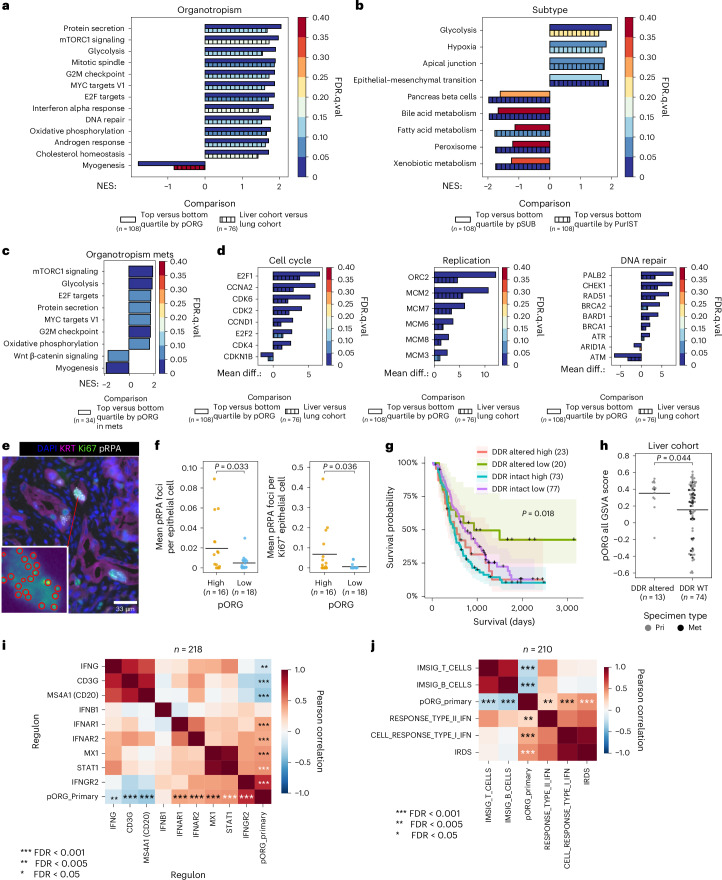
High pORG, liver-tropic PDAC is associated with replication stress tolerance and IFN response. **a**–**c**, NES colored by FDR *P*-adjusted (FDR.q) value (FDR.q.val) (from one-way ANOVA) is shown for Hallmark GSEA pathways if any of the comparisons reached a NES > 1.7 and FDR.q < 0.05 from the cohorts indicated on each plot. **a**, Solid bars, top versus bottom quartile by pORG (*n* = 108 pts.); hatched bars, liver versus lung cohort (*n* = 76 pts.). **b**, Solid bars, top versus bottom quartile by pSUB (*n* = 108 pts.); hatched bars, top versus bottom quartile by PurIST (*n* = 108 pts.). **c**, Solid bars, top versus bottom quartile by pORG in metastases (mets) (*n* = 34 pts.). **d**, Mean differential (diff.) VIPER regulon activity scores colored by FDR.q.val (from one-way ANOVA) in top versus bottom quartile by pORG (solid bars, *n* = 108 pts.) and liver cohort versus lung cohort (hatched bars, *n* = 76 pts.) primary tumors for regulons related to cell cycle (left), DNA replication (center) and DNA damage repair (right). **e**, Example immunostaining of epithelial cells (KRT^+^), proliferation (Ki67^+^) and algorithmic detection of pRPA foci in PDAC tissue (*n* = 55 cores imaged in total). **f**, Mean RS pRPA foci in epithelial cells (left; high pORG (*n* = 16 pts.), low pORG (*n* = 18 pts.), *P* = 0.033) and Ki67^+^ proliferating epithelial cells (right; high pORG (*n* = 16 pts.), low pORG (*n* = 18 pts.), *P* = 0.036) in each patient determined by immunostaining a TMA with 34 primary specimens, 1–2 cores each. **g**, K–M estimate of OS for patients with tumors with high or low pORG GSVA scores stratified by tumors with or without a known pathologic somatic alteration (VUS were excluded) in a DDR-related gene (DDR altered high (*n* = 23 pts.), DDR intact high (*n* = 73 pts.), DDR altered low (*n* = 20 pts.) or DDR intact low (*n* = 77 pts.), *P* = 0.018). log-rank *P* value, and shaded regions represent 95% CI. **h**, pORG GSVA scores for primary tumors (Pri) and metastases (Met) from patients in the liver cohort categorized by a known pathologic somatic alteration (VUS were excluded) in a DDR-related gene (DDR altered (*n* = 13 tumors) or DDR WT (*n* = 74 tumors), *P* = 0.044). **i**, Pearson correlation (two-sided) of the indicated VIPER regulon scores and pORG GSVA scores (*n* = 218 pts.). **j**, Pearson correlation (two-sided) of pORG and IFN- and immune-related signature GSVA scores for primary tumors (*n* = 210 pts.). Two-tailed Students *t*-test *P* value; black bars represent the mean (**f**,**h**). *P* values from Pearson correlation and corrected with the Benjamini–Hochberg method (**i**,**j**). *FDR *P* adjusted < 0.05, **P adjusted < 0.01, ***P adjusted < 0.001. FDR-adjusted *P* values were 0.0037, 4.7 × 10^−6^, 3.5 × 10^−8^, 0.24, 1.8 × 10^−9^, 2.6 × 10^−8^, 5 × 10^−11^, 8 × 10^−12^, 7.4 × 10^−39^, *n* = 218 patients (for **i**) and 8.8 × 10^−5^, 1.8 × 10^−5^, 0.0047, 2 × 10^−7^, 4.1 × 10^−11^ (for **j**), *n* = 210 patients. Source data

**Extended Data Fig. 5:**
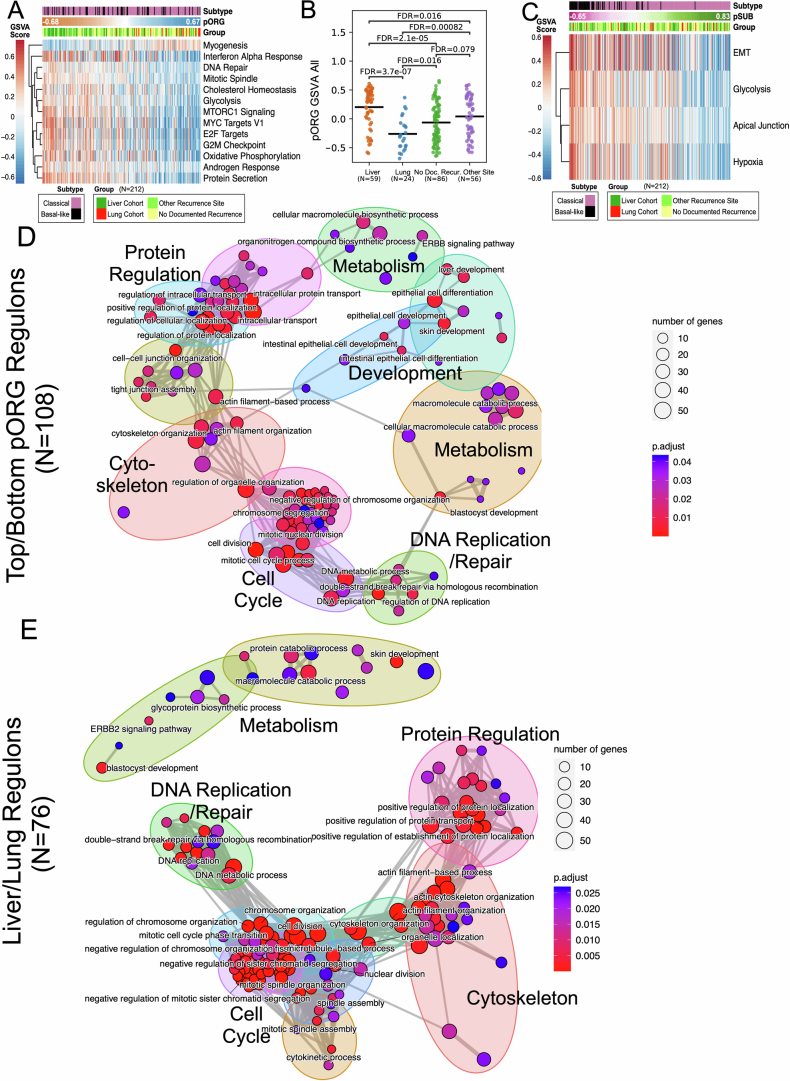
Organotropism and subtype association with recurrence site and biological processes. **A)** Heatmap of GSVA scores of MSigDB “Hallmark” gene sets with NES > 1.7 and FDR.Q < 0.05 by GSEA for top versus bottom quartile by pORG. Primary tumor samples (columns) are ordered from highest-to-lowest by pORG score. N = 212 pts. **B)** pORG score of primary tumors in liver, lung, other site or no documented recurrence metastatic cohorts that is liver or lung (N = 59 and N = 24, FDR = 3.7e-07), liver or no documented recurrence (N = 59 and N = 86, FDR = 2.1e-05), liver or other site recurrence (N = 59 and N = 56, FDR = 0.016), lung or no documented recurrence (N = 24 and N = 86, FDR = 0.016), lung or other site recurrence (N = 24 and N = 56, FDR = 0.00082), and no documented recurrence or other site recurrence (N = 86 and N = 56, FDR = 0.079). P values obtained from two-tailed t-test and corrected with the Benjamini/Hochberg method. Black bars represent means. N=number of patients. **C)** Heatmap of Hallmark GSVA scores with NES > 1.7 and FDR.Q < 0.05 by GSEA for top versus bottom quartile by pSUB. Primary tumor samples (columns) are ordered from highest-to-lowest by pSUB score. N = 212 pts. **D-E)** Enrichment map depicting Gene Ontology (GO) biological process enrichment results for the top 300 VIPER regulons significantly increased (by one-way ANOVA; FDR.Q-value ≤ 0.05) in **D)** top pORG primary tumors compared to bottom pORG (N = 108 pts.), or **E)** liver cohort primary tumors compared to lung cohort (N = 76 pts.). Top regulons were selected by order of greatest increase in mean regulon score for (**D**) top pORG samples or (**E**) liver cohort samples. Up to 150 enriched GO terms (points) are shown, but only the top 3 most significant terms within each cluster (large colored circles) are labeled. GO terms were arranged into clusters based on their semantic similarity. Point size scales with the number of regulons annotated with the indicated GO term. Edges connecting GO terms indicate a Jaccard similarity of at least 0.2 (scaled by width). Source data

Gene set enrichment analysis (GSEA) analysis revealed that high pORG and liver cohort primary tumors were enriched (normalized enrichment score (NES) > 1.7, false discovery rate (FDR) < 0.05) in Hallmark pathways^26^ related to oncogene-mediated RS: G2M checkpoint, E2F targets, mitotic spindle, MYC targets V1, DNA repair, IFN-α response, cell metabolism and mitogenesis (Fig. 3a). We found that high pSUB and PurIST primary tumors were enriched in pathways related to glycolysis, epithelial–mesenchymal transition, apical junctions and hypoxia, whereas high PurIST was de-enriched for bile acid metabolism and pancreas β cells (Fig. 3b). Visualization of GSVA scores for these pathways supported results from GSEA and showed that the no documented recurrence clinical group skewed pORG low (Extended Data Fig. 5a–c). GSEA in metastatic-sample cohorts yielded significant differences only in high versus low pORG, with 7 upregulated pathways overlapping with the 12 found in primaries (Fig. 3c). Thus, separating primary tumor metastatic organotropism and molecular subtype using the pORG and pSUB gene sets identifies unique pathway enrichments.

### Cell cycle, RS and DNA repair up in high pORG, liver-avid tumors

**Extended Data Fig. 6:**
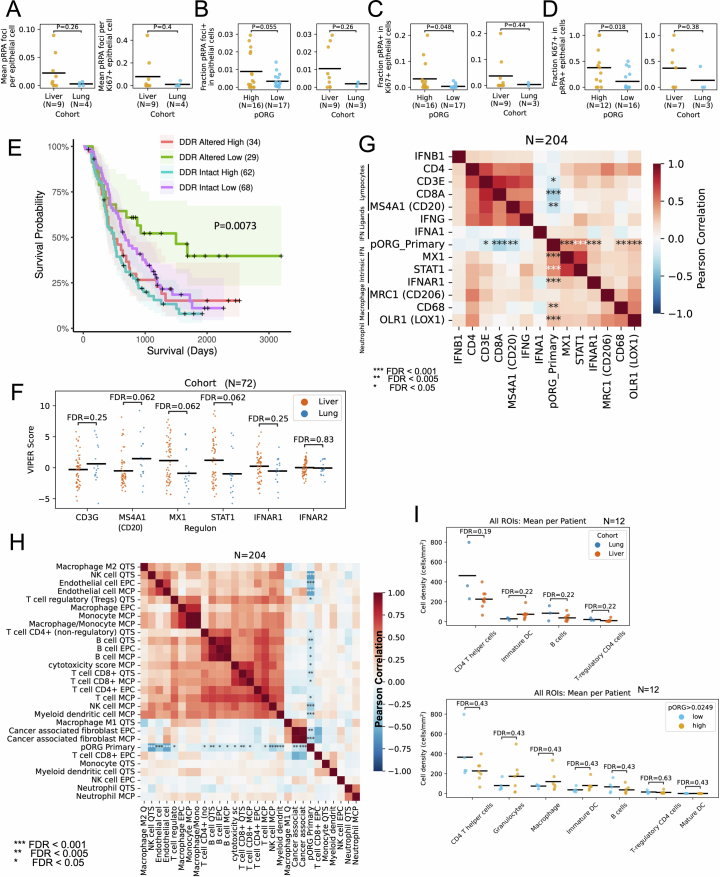
Relationship of organotropism to replication stress, tolerance of DDR-pathway alteration and immune infiltration. **A)** pRPA foci per epithelial cell (left, liver [N = 9], lung [N = 4, P = 0.26]) and per Ki67+ epithelial cell (right, liver [N = 9], lung [N = 4, P = 0.4]) in liver versus lung cohort primary tumors on the TMA. **B)** Percent of epithelial cells positive for pRPA foci for pORG high or low (N = 16 and N = 17, P = 0.055) and liver versus lung cohort (N = 9 and N = 3, P = 0.26) **C)** Percent of proliferating epithelial cells positive for pRPA foci for pORG high or low (N = 16 and N = 17, P = 0.048) and liver versus lung cohort (N = 9 and N = 3, P = 0.44). **D)** Percent of pRPA+ epithelial cells that are proliferating for pORG high or low (N = 12 and N = 16, P = 0.018) and liver versus lung cohort (N = 7 and N = 3, P = 0.38). **E)** Kaplan–Meier estimates of overall survival for patients with tumors with high or low pORG GSVA scores stratified by tumors with or without a non-silent somatic alteration in a DDR-related gene that is DDR altered high (N = 34), DDR altered low (N = 29), DDR intact high (N = 62), and DDR intact low (N = 68, P = 0.0073). P value determined by log-rank test and shaded regions represent 95% confidence intervals. **F)** VIPER regulon scores in liver versus lung cohort for genes CD3G (N = 72, FDR = 0.25), MS4A1 (N = 72, FDR = 0.062), MX1 (N = 72, FDR = 0.062), STAT1 (N = 72, FDR = 0.062), IFNAR1 (N = 72, FDR = 0.25) and IFNAR2 (N = 72, FDR = 0.82). **G)** Pearson correlation of marker gene expression from RNA-seq and pORG GSVA score for primary tumors (Exact FDR values are 0.24, 0.43, 0.026, 5.9e-06, 0.0018, 0.44, 0.11, na, 1.9e-09, 6.6e-11, 0.0008, 0.24, 0.0018, 6.4e-05, N = 204). **H)** Pearson correlation of deconvolution algorithm scores and pORG GSVA score for primary tumors. Deconvolution algorithms include QTS: quanTIseq; MCP: MCP-counter; EPC: EPIC (see Methods, exact FDR values are 0.062, 7e-20, 1.2e-08, 2.2e-17, 0.012, 0.7, 0.1, 0.1, 0.012, 0.0017, 0.0053, 0.01, 0.025, 0.005,0.034, 0.1, 0.012, 2.5e-07, 0.0008, 0.81, 0.0029, 0.0001, na, 0.84, 0.095, 0.68, 0.45, 0.065, 0.45, N = 204). **I)** mIHC mean cell density per patient for each indicated cell type that is CD4 T helper cells (N = 12, FDR = 0.19), immature DC (N = 12, FDR = 0.22), B cells (N = 12, FDR = 0.22), and T regulatory CD4 cells (N = 12, FDR = 0.22) significant at the ROI cohort level in lung versus liver cohort primaries (top) and low versus high pORG score primaries (bottom) that is CD4 T helper cells N = 12, FDR = 0.43), granulocytes (N = 12, FDR = 0.43), Macrophage (N = 12, FDR = 0.43), immature DC (N = 12, FDR = 0.43), B cells (N = 12, FDR = 0.43), T-regulatory. Each dot represents a patient. **A-D)** Each data point is the average across two primary tumor TMA cores imaged per patient in high and low pORG or liver and lung cohort groups. P values from two-tailed t-test. Black bars represent the means. N=number of patients. **F, I)** P values from two-tailed t-test corrected with Benjamini/Hochberg method. Black bars represent the means. N=number of patients. **G, H)** FDR corrected P values from two-sided Pearson correlation with pORG GSVA score, *** FDR < 0.001 ** FDR < 0.005 * FDR < 0.05. N=number of patients. Source data

Virtual inference of protein-activity enrichment regulon (VIPER)^27,28^ analysis followed by Gene Ontology network analyses identified nodes for cell cycle and DNA replication and repair enriched in both high pORG and liver cohort primary tumors (Extended Data Fig. 5d,e). Accordingly, cell cycle, DNA replication and DNA repair proteins demonstrated significantly higher activity in high pORG and liver cohort tumors (Fig. 3d). To further analyze RS, we immunostained a tissue microarray (TMA) prepared from 34 primary tumors using the same FFPE blocks from our RNA and DNA-seq data for foci of phosphorylated replication protein A (pRPA) (Fig. 3e), an indicator of single-stranded DNA exposed during RS. We found a significantly higher mean number of pRPA foci in cytokeratin-positive (KRT^+^) epithelial cells in high pORG versus low pORG primary tumors (Fig. 3f, left). Additionally, we found that Ki67^+^ proliferating tumor cells had significantly more pRPA foci in high pORG tumors (Fig. 3f, right). Similar, though not significant, trends were observed in nine liver cohort primary tumors compared to four lung cohort (Extended Data Fig. 6a). The percent of epithelial cells and of proliferating epithelial cells positive for pRPA foci were also higher in high pORG primary tumors and trended higher in liver cohort primaries (Extended Data Fig. 6b-c), and more pRPA^+^ cells were proliferating in high pORG tissues (Extended Data Fig. 6d). Together, these results support the hypothesis that in high pORG tumors, pRPA^+^ cells are a viable, expanding part of the tumor despite ongoing RS, likely due to the associated increase in DNA repair.

### Low pORG tumors are less tolerant to defects in DNA repair

A report by Dreyer et al. suggested that treatment-agent efficacy may depend on both RS and DNA damage response (DDR) gene alteration status, dividing patients into four categories based on the presence or absence of those two factors^16^. As our data indicate that liver-avid, high pORG primary tumors are enriched for pathways associated with ongoing RS and DNA repair, we divided patients into four categories by high/low pORG score and the presence/absence of a known DDR gene alteration^29^. Although patients with high pORG scoring tumors fared poorly regardless of DDR gene status, patients with low pORG tumor scores survived significantly longer if their primary tumors had DDR nonsilent gene alterations, whether or not variants of unknown significance (VUS) were excluded (Fig. 3g and Extended Data Fig. 6e). Additionally, liver cohort tumors with DDR gene alterations had higher pORG scores compared to those without (Fig. 3h), suggesting that the presence of DDR gene alterations may promote mechanisms supporting tumor cell responses to RS and DNA damage to avoid mitotic catastrophe, and a lack of this response, as seen in low pORG tumors, combined with a DDR gene alteration improves patient outcome (Fig. 3g).

### Suppressed tumor immunity in high pORG, liver-avid tumors

Consistent with enrichment of the Hallmark IFN-α response in high pORG samples by GSEA (Fig. 3a), VIPER scores for IFN-α/β receptor subunits activity positively correlated with pORG score (Fig. 3i). Chronic IFN signaling in cancer is reported to induce an IFN-related DNA damage resistance gene expression signature (IRDS), associated with tumor cell resistance to DNA damage^30–32^ and escape from tumor immunity^33^. We found a significant positive correlation between the IRDS gene signature and pORG score in primary tumors (Fig. 3j). Two genes in the IRDS gene set matched VIPER regulons (STAT1 and MX1) and these were both significantly positively correlated with pORG and trended higher in liver cohort tumors (Fig. 3i and Extended Data Fig. 6f).

**Fig. 4:**
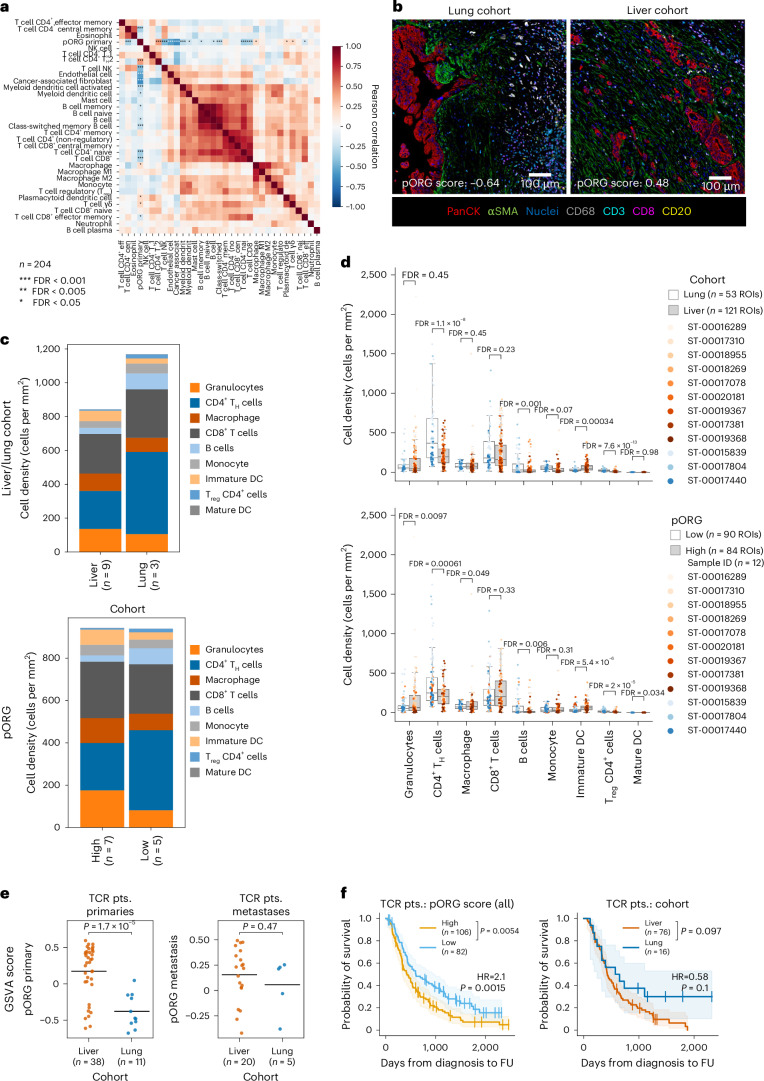
Transcriptomic and multiplex imaging evidence of immune suppression in high pORG, liver-tropic tumors. **a**, Pearson correlation (two-sided) of xCell deconvolution scores and pORG GSVA score for primary tumors (*n* = 204 pts.). *P* values from Pearson correlation and corrected with the Benjamini–Hochberg method. *FDR *P* adjusted < 0.05, **P adjusted < 0.01, ***P adjusted < 0.001. FDR-corrected *P* values are 0.198, 9.06 × 10^−6^, 0.0752, 0.802, 0.123, 2.31 × 10^−9^, 5.23 × 10^−10^, 1.26 × 10^−16^, 2.58 × 10^−22^, 0.000701, 0.0112, 0.164, 0.0215, 0.0966, 0.0125, 0.000937, 0.0682, 0.281, 0.326, 1.93 × 10^−6^, 3.54 × 10^−5^, 0.0215, 0.362, 0.228, 0.462, 0.326, 0.0147, 0.00857, 0.362, 0.018, 0.422 and 0.227. **b**, Representative images of mIHC staining of a low pORG, lung cohort patient tumor (left) and a high pORG, liver cohort patient tumor (right). *n* = 12 tissues imaged, 174 ROIs total. **c**, Average leukocyte densities for primary tumors from patients in the liver cohort (mean pORG 0.23 s.e.m. = 0.11, *n* = 9 pts.) and lung cohort (mean pORG −0.51 s.e.m. = 0.09, *n* = 3 pts.) (top). Average leukocyte densities for primary tumors from patients with high pORG (pORG 0.38 s.e.m. = 0.04, *n* = 7 pts.) and low pORG GSVA scores (mean pORG –0.43 s.e.m. = 0.08, *n* = 5 pts.) (bottom). DC, dendritic cell. **d**, Leukocyte densities in ROIs from liver (*n* = 121 ROIs) or lung cohort (*n* = 53 ROIs) primaries (top) and high (*n* = 84 ROIs) or low (*n* = 90 ROIs) pORG primaries (bottom). Each dot represents an ROI colored by patient specimen (*n* = 12 patients). Box represents the median and interquartile range (IQR), and whiskers extend 1.5 × IQR. *P* values from two-tailed *t*-test corrected with the Benjamini–Hochberg method. FDR-corrected *P* values are 0.45, 1.1 × 10^−8^, 0.45, 0.23, 0.001, 0.07, 0.00034, 7.6 × 10^−13^, 0.98 (top) and 0.0097, 0.00061, 0.049, 0.33, 0.006, 0.31, 5.4 × 10^−6^, 2 × 10^−5^, 0.034 (bottom, *n* = 174 ROIs). **e**, pORG score from RNA-seq of liver versus lung cohort tumors in the TCRβ dataset, primaries (left; liver (*n* = 38 pts.), lung (*n* = 11 pts.), *P* = 1.7 × 10^−5^) and metastases (right: liver (*n* = 20 pts.), lung (*n* = 5 pts.), *P* = 0.47). *P* values from two-tailed *t*-test. Black bars represent the means. **f**, K–M estimation of OS of patients with high (*n* = 106 pts.) versus low (*n* = 82 pts.; *P* = 0.0054) pORG GSVA scores (left; cutoff determined by ROC and maximum Youden’s index in the full dataset in Fig. 2a) and liver (*n* = 76 pts.) versus lung cohort (*n* = 16 pts.; *P* = 0.097) patients (right) in the TCRβ dataset. log-rank test *P* values and *n* patients per group are indicated with brackets and shaded regions represent 95% CI. CPH single-variable modeling HR and associated *P* values are displayed on plots. Source data

Consistent with chronic IFN signaling inactivating adaptive immune cells^34^, we found that high pORG scores negatively correlated with B cell and T cell gene signatures, regulons and marker genes; and positively correlated with response to IFN, macrophage and neutrophil marker genes, signatures, and regulons (Fig. 3i,j and Extended Data Fig. 6g). Similarly, liver versus lung cohort tumors had a trend of lower CD20 B cell VIPER activity scores (FDR = 0.06; Extended Data Fig. 6f). We found similar results with deconvolution algorithms: notably, negative correlations between pORG score and most lymphocyte subsets, as well as endothelial cells and cancer-associated fibroblasts, and positive correlations between pORG score and immune suppressive T_H_2 CD4^+^ T cells, macrophages, plasmacytoid dendritic cells and γδ T cells (Fig. 4a and Extended Data Fig. 6h).

We used a multiplexed immunohistochemistry (mIHC) platform^35,36^ to measure densities of leukocyte subsets in multiple 1.0-mm^2^ regions of interest (ROIs) in tissue sections from primary tumor specimens with pORG scores assigned from gene expression data and classified as the liver (121 ROIs, *n* = 9 patients) or lung cohort (53 ROIs, *n* = 3 patients; Fig. 4b). Seven of the nine liver cohort samples were scored high pORG (84 ROIs); and the five low pORG samples included the three lung cohort samples and two liver cohort samples (90 ROIs). Consistent with leukocyte-relevant gene expression, at the ROI cohort level, we found that the low pORG and lung cohort tumors harbored greater densities of CD4^+^ T helper cells, B cells and T regulatory CD4^+^ cells, whereas the high pORG tumors had higher granulocytes, macrophages and immature dendritic cells, which were also enriched in the liver cohort (Fig. 4c,d). With the low sample numbers, these were not significant at the patient-level comparisons (Extended Data Fig. 6i), and additional samples will need to be evaluated by mIHC to validate these findings. Taken together, these data demonstrate that aggressive, high pORG liver-avid primary PDAC tumors are characterized by both ongoing RS response and likely evasion of antitumor immunity.

### T cell repertoires are rich and diverse in low pORG tumors

**Extended Data Fig. 7:**
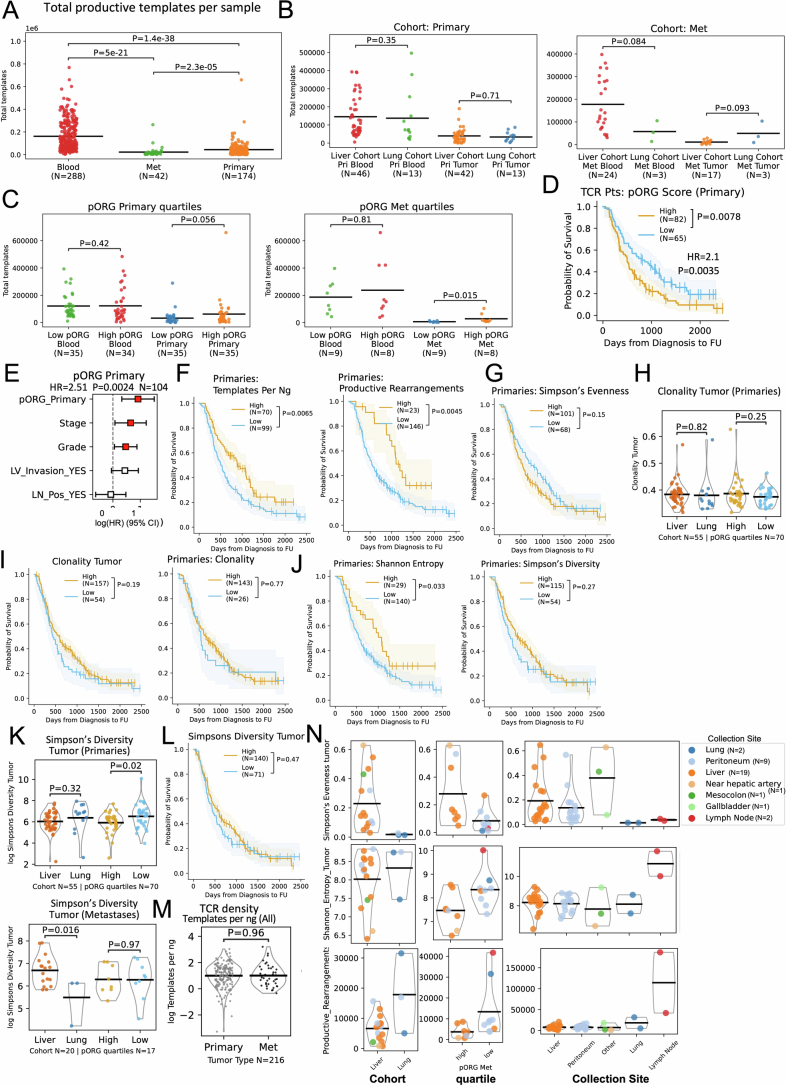
Association of tumoral TCR sequences and organotropism, patient outcome or recurrence site. **A-C)** Total productive TCRβ templates sequenced per patient in **A)** blood samples or samples from primary or metastatic tumors (blood versus primary [N = 288 and N = 174, P = 1e-38], blood versus met [N = 289 and N = 42, P = 4.7e-21] and primary versus met [N = 174 and N = 42, P = 2.3e-05]) or blood or tumor samples from **B)** primary liver or lung cohort (left, liver versus lung blood [N = 46 and N = 13, P = 0.35], liver versus lung tumor [N = 42 and N = 13, P = 0.71]) or metastatic liver or lung cohort (right, liver versus lung blood [N = 24 and N = 3, P = 0.084], liver versus lung tumor [N = 17 and N = 3, P = 0.093]) or **C)** high or low pORG primary (left, low versus high pORG blood [N = 35 and N = 34, P = 0.42], low versus high pORG primary [N = 35 and N = 35, P = 0.056]) or metastatic (right, low versus high pORG blood [N = 9 and N = 8, P = 0.81], or low versus high pORG met [N = 9 and N = 8, P = 0.015]) cohort samples. Primary tumors and blood collected during primary resectable disease and metastatic tumors and blood collected during metastatic disease. **D)** Kaplan–Meier (K–M) estimation of overall survival (OS) in primary tumor sampled patients in the TCRβ dataset with high (N = 82) versus low (N = 65, P = 0.0078) pORG GSVA scores. **E)** Cox proportional hazards multi-variable modeling of OS versus TCRβ dataset patient’s primary tumor pORG GSVA score with clinical covariates (N = 104, P = 0.0024). HR and associated P value for variable in bold was determined by CPH modeling. Hazard ratios indicated by boxes and 95% confidence intervals by error bars. **F-G)** K–M estimate of OS of patients with high versus low **F)** templates per nanogram (ng) (left, high [N = 70] or low [N = 99, P = 0.0065]) or productive rearrangements (right, high [N = 23] or low [N = 146, P = 0.0045]) or **G)** Simpson’s evenness in primary tumors (high [N = 101] or low [N = 68, P = 0.15]). **H)** Tumor TCRβ Clonality in high/low pORG (N = 70, P = 0.25) or liver/lung (N = 55, P = 0.82) cohorts in primaries. **I)** K–M estimate of OS of patients with high versus low Clonality in all tumors (left, high [N = 157] or low [N = 54, P = 0.19]) and primary tumors (right, [N = 143] or low [N = 26, P = 0.77]). **J)** K–M estimate of OS of patients with high versus low TCRβ Shannon entropy (left, high [N = 29] or low [N = 140, P = 0.033]) and Simpson’s diversity (right, high [N = 115] or low [N = 54, P = 0.27]) in primary tumors. **K)** TCRβ Simpson’s diversity in high/low pORG or liver/lung cohorts in primary tumors (top, high or low pORG [N = 70, P = 0.02], liver or lung [N = 55, P = 0.32]) or metastases (bottom, high or low pORG [N = 17, P = 0.97], liver or lung [N = 20, P = 0.016]). **L)** K–M estimate of OS of patients with high (N = 140) versus low (N = 71, P = 0.47) TCRβ Simpson’s diversity. **M)** Templates per nanogram (ng) in all primary tumors and metastases (N = 216, P = 0.96). **N)** The indicated TCR metrics in metastatic tumors colored by collection site of metastasis that is lung (N = 2), peritoneum (N = 9), liver (N = 19), near hepatic artery (N = 1), mesocolon (N = 1), gallbladder (N = 1), or lymph node (N = 2), grouped by liver versus lung cohort (left), high versus low pORG quartiles (center), and metastatic collection site based on surgical and radiologic notes (right). Black bars represent the means **A-C)** P values from two-tailed Mann-Whitney U test, black bars represent the means. N=number of patients. **D, F-G, I-J, L)** High/low cutoff determined with the max Youden index. P values between groups indicated with brackets determined by log-rank test and shaded regions represent 95% confidence intervals. Patients who died within 30 days after resection are not shown. N=number of patients. **H, K, M)** P values from one-way ANOVA, black bars represent the means. N=number of patients. Source data

We performed sequencing of genomic rearrangements encoding the complementarity determining region 3 (CDR3) of TCRβ chains from 288 blood samples and 216 tumors (174 primary and 42 metastatic), 215 of which had matched blood from the same patient. RNA-seq was available for 175 patients with TCR-seq analysis of tumor, and of these, 139 patients had their primary tumor analyzed and 33 patients had their metastatic tumor analyzed with both modalities (Extended Data Fig. 1e). Seventy-six blood samples were from patients in the liver cohort and 16 were from patients in the lung cohort, of which 59 and 16 were matched with tumor samples from the same patient. The number of productive templates sequenced were highest in blood samples, and lower in metastases, compared to primary tumors, but there was little-to-no significant difference within a sample type in our cohort comparisons: liver versus lung and high versus low pORG (Extended Data Fig. 7a–c). Liver and lung cohort primary tumors in the TCR-seq dataset with RNA-seq were significantly separated by pORG score, but not the metastases (Fig. 4e). Patient outcomes for the TCR-seq dataset matched those of the whole cohort for pORG (Fig. 4f and Extended Data Fig. 7d,e); but lung cohort patient survival only trended longer, potentially due to the relatively low number of lung cohort patients in the TCR-seq dataset (Fig. 4f).

**Fig. 5:**
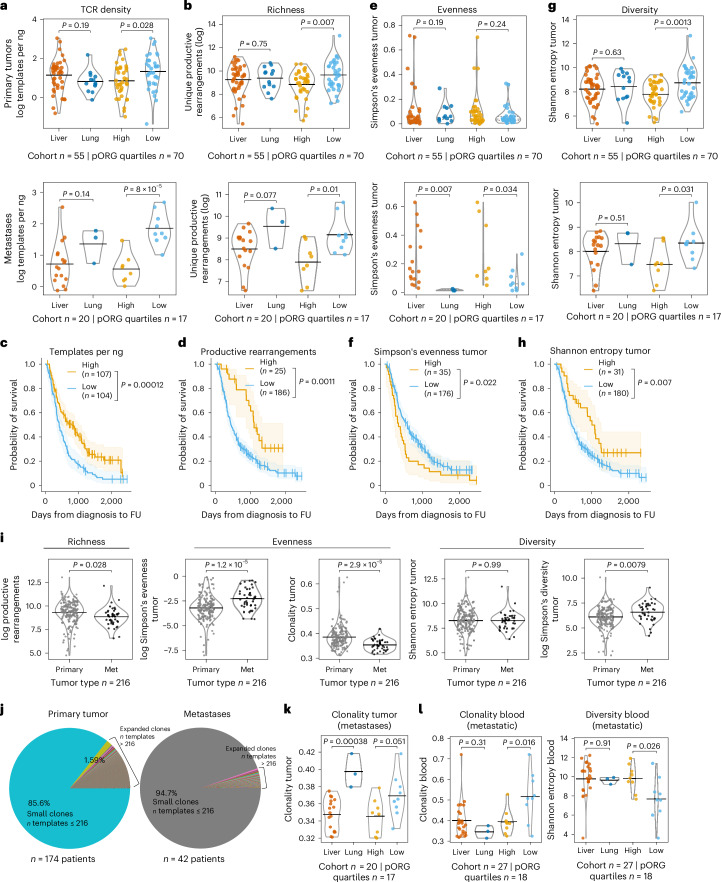
Tumoral TCRβ repertoire richness, diversity associated with low pORG tumors; clonality decreases in metastases, but not in low pORG, lung metastases. **a**, TCRβ templates per ng of DNA sequenced in primary tumors (top; liver or lung (*n* = 55 pts., *P* = 0.19), pORG quartiles high or low (*n* = 70 pts., *P* = 0.028)) and metastases (bottom; liver or lung (*n* = 20 pts., *P* = 0.14), pORG quartiles high or low (*n* = 17 pts., *P* = 8 × 10^−5^)) in liver versus lung and high (top quartile) versus low (bottom quartile) pORG tumors. **b**, The number of unique productive rearrangements of TCRβ templates in primary tumors (top; liver or lung (*n* = 55 pts., *P* = 0.75), high or low (*n* = 70 pts., *P* = 0.007)) and metastases (bottom; liver or lung (*n* = 20 pts., *P* = 0.077), high or low (*n* = 17 pts., *P* = 0.01)), grouped by the indicated cohorts. **c**,**d**, K–M estimates of OS of patients with high versus low templates per ng (**c**) (high (*n* = 107 pts.) or low (*n* = 104 pts.), *P* = 0.00012) and productive rearrangements (**d**) in all tumors (high (*n* = 25 pts.) or low (*n* = 186 pts.), *P* = 0.0011). **e**, Simpson’s evenness estimation of TCRβ repertoire evenness in primary tumors (top; liver or lung (*n* = 55 pts., *P* = 0.19), high or low (*n* = 70 pts., *P* = 0.24)) and metastases (bottom; liver or lung (*n* = 20 pts., *P* = 0.007), high or low (*n* = 17 pts., *P* = 0.034)), grouped by the indicated cohorts. *P* values from Kruskal–Wallis H-test; dashed lines represent the median and IQR. **f**, K–M estimates of OS of patients with high (*n* = 35 pts.) versus low (*n* = 176 pts.; *P* = 0.022) Simpson’s evenness. **g**, Shannon entropy estimation of TCRβ repertoire diversity in primary tumors (top; liver or lung (*n* = 55 pts., *P* = 0.63), high or low (*n* = 70 pts., *P* = 0.0013)) and metastases (bottom; liver or lung (*n* = 20 pts., *P* = 0.51), high or low (*n* = 17 pts., *P* = 0.031)), grouped by the indicated cohorts. **h**, K–M estimates of OS of patients with high (*n* = 31 pts.) versus low (*n* = 180 pts.; *P* = 0.007) Shannon entropy. **i**, The indicated TCR metrics in metastases (Met) versus primary tumors grouped by related TCR metrics; productive rearrangement (*n* = 216 pts., *P* = 0.028), Simpson’s evenness estimation (*n* = 216 pts. *P* = 1.2 × 10^−5^), clonality (*n* = 216 pts., *P* = 2.9 × 10^−5^), Shannon entropy estimation (*n* = 216 pts., *P* = 0.99) and Simpson’s diversity estimation (*n* = 216 pts., *P* = 0.0079). **j**, Pie charts of fraction of each CDR3 sequence in TCRβ repertoires of primary tumors (*n* = 174 pts.) or metastases (*n* = 42 pts.). The largest slice is all the small clones (those present in less than or equal to one template per patient on average in tumor samples) and each smaller slice of pie is an expanded clone present at greater than one template per patient on average across the tumors. **k**, Tumor TCRβ clonality in high/low (*n* = 17 pts., *P* = 0.051) pORG or liver/lung cohorts (*n* = 20 pts., *P* = 0.00038) in metastases. **l**, Clonality (left; liver or lung (*n* = 27 pts., *P* = 0.31), high or low (*n* = 18 pts., *P* = 0.016)) and Simpson’s diversity (right; liver or lung (*n* = 27 pts., *P* = 0.91), high or low (*n* = 18 pts., *P* = 0.026)) in bloods collected from patients with metastases, grouped by the indicated cohorts. High/low cutoff determined with the ROC and maximum Youden’s index for each metric, log-rank *P* value and *n* per group shown with bracket, and shaded regions represent 95% CI (**c**,**d**,**f**,**h**). Patients who died within 30 days after resection are not shown. *P* values were derived from a one-way ANOVA; black bars represent the mean (**a**,**b**,**g**,**i**,**k**,**l**). Source data

We evaluated T cell repertoires using common metrics of richness (the number of unique TCRβ CDR3 amino acid sequences), evenness (the distribution of clonal frequencies within a sample; a very clonal repertoire would have low evenness) and diversity (a function of both richness and evenness)^37,38^. We applied these metrics in the context of tumor type (primary versus metastatic), pORG score and liver versus lung cohort; moreover, we examined the influence of each repertoire metric on OS, across all patients and primary tumor sampled patients in the TCR-seq dataset. Consistent with greater T cell enrichment in low pORG tumors (Figs. 3i,j and 4a–d and Extended Data Fig. 6g,h), we found a higher density of productive TCRβ templates (templates per ng) and more unique productive TCRβ rearrangements (richness) in low pORG primary and metastatic tumors relative to high pORG (Fig. 5a,b). We did not detect the same difference between lung and liver cohort tumors, although lung and liver cohort metastases trended similarly (Fig. 5a,b). Patient survival time increased with greater TCRβ template density or richness (Fig. 5c,d and Extended Data Fig. 7f). These data suggest that the underlying biology associated with a high pORG signature may restrict the density of T cells in the tumor and reduce the richness of the TCR repertoire.

TCR clonal distribution was evaluated with two evenness metrics: Simpson’s evenness and Pielou evenness (also known as richness-normalized Shannon entropy), which expressed as 1 − Pielou evenness is termed clonality^39,40^. We found lower Simpson’s evenness in low pORG and lung cohort tumors relative to high pORG and liver cohort tumors, trending in primary tumors and significant in metastases (Fig. 5e); and low Simpson’s evenness was associated with better patient outcomes (Fig. 5f and Extended Data Fig. 7g). Consistent with low Simpson’s evenness, clonality was higher in lung cohort metastases and trended higher in low pORG metastases (Extended Data Fig. 7h), but the overall outcome trend was not significant (Extended Data Fig. 7i).

We used Shannon entropy and Simpson’s diversity (1 − Simpson’s *d*) to evaluate TCR repertoire diversity^38,40,41^. Shannon entropy is maximized with increasing richness and increasing evenness of the TCR sequences, while Simpson’s diversity de-emphasizes low-frequency clones and is thus less affected by richness. We observed that low pORG primary and metastatic tumors have high Shannon entropy (Fig. 5g), which is associated with better patient outcome (Fig. 5h and Extended Data Fig. 7j). Similar to Shannon entropy, Simpson’s diversity was higher in low pORG primaries; however, it was not significantly associated with patient survival (Extended Data Fig. 7j–l), indicating high diversity in low-frequency TCR clones is more strongly associated with patient outcomes (Fig. 5h and Extended Data Fig. 7j). Lung cohort relative to liver cohort tumors did not have increased TCR Shannon entropy (Fig. 5g); instead, lung cohort metastases had low Simpson’s diversity (Extended Data Fig. 7k), which is consistent with their high clonality (Fig. 5k).

### Low pORG/lung cohort metastases maintain higher TCR clonality

**Extended Data Fig. 8:**
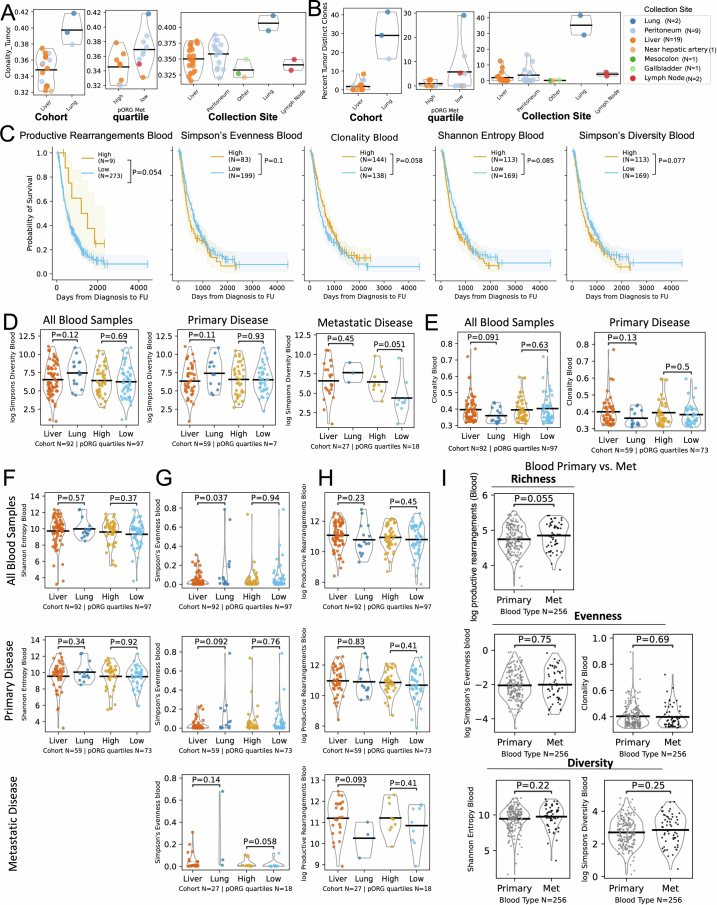
Association of blood TCR sequences and organotropism, patient outcome or disease progression. **A-B)**. Metastatic tumors’ TCRβ repertoire **A)** Clonality and **B)** tumor-distinct clones that is, found in tumor but not matched blood, colored by collection site of metastasis, i.e lung (N = 2), peritoneum (N = 9), liver (N = 19), near hepatic artery (N = 1), mesocolon (N = 1), gallbladder (N = 1), or lymph node (N = 2), grouped by liver versus lung cohort (left), high versus low pORG quartiles (center), and metastatic collection site based on surgical and radiologic notes (right). **C)** Kaplan–Meier estimate of overall survival of patients with high versus low TCR metrics in blood samples that is productive rearrangement (high [N = 130] or low [N = 62, P = 0.02]), Simpson’s evenness (high [N = 83], or low [N = 199, P = 0.1]), clonality (high [N = 144], or low [N = 138, P = 0.058]), Shannon entropy (high [N = 113], or low [N = 169, P = 0.085]) and Simpson’s diversity (high [N = 113], or low [N = 169, P = 0.077]). High/low cutoff determined with the ROC max Youden index. P values between groups indicated with brackets determined by log-rank test and shaded regions represent 95% confidence intervals. Patients who died within 30 days after resection are not shown. N=number of patients. **D)** TCR Simpson’s diversity (1- Simpson’s D) in all (left, liver or lung [N = 92, P = 0.12], high or low pORG quartiles [N = 97, P = 0.69)), primary- associated (center liver or lung [N = 59, P = 0.11], high or low pORG quartiles [N = 7, P = 0.93]), and metastatic blood (right, liver or lung [N = 27, P = 0.45], high or low pORG quartiles [N = 18, P = 0.051])**.E)** TCR Clonality (that is 1 – Normalized Shannon Entropy) in all (left, liver or lung [N = 92, P = 0.091], high or low pORG quartiles [N = 97, P = 0.63]) and primary-associated blood (right, liver or lung [N = 59, P = 0.13], high or low pORG quartiles [N = 73, P = 0.5]) **F-H)** TCR metrics in all blood samples (top), blood collected from patients at the time of primary resectable disease (center) and collected during metastatic disease (bottom) from the indicated cohorts. Metrics are **F)** Shannon entropy in all blood samples (top, liver or lung [N = 92, P = 0.57], high or low pORG quartiles [N = 97, P = 0.37]), and at time of primary resectable disease (center, liver or lung [N = 59, P = 0.34], high or low pORG quartiles [N = 73, P = 0.92]).**G)** Simpson’s evenness in all blood samples (top, liver or lung [N = 92, P = 0.037], high or low pORG quartiles [N = 97, P = 0.94]), at time of primary resectable disease (center, liver or lung [N = 59, P = 0.092], high or low pORG quartiles [N = 73, P = 0.76]), and during metastatic disease (bottom, liver or lung [N = 27, P = 0.14], high or low pORG quartiles [N = 18, P = 0.058]). **H)** (log) number of unique productive rearrangements of TCRβ templates in all blood samples (top, liver or lung [N = 92, P = 0.23], high or low pORG quartiles [N = 97, P = 0.45]), at time of primary resectable disease (center, liver or lung [N = 59, P = 0.83], high or low pORG quartiles [N = 73, P = 0.41]), and during metastatic disease (bottom, liver or lung [N = 27, P = 0.093], high or low pORG quartiles [N = 18, P = 0.41) **I)** TCR metrics for richness (N = 256, P = 0.055), evenness that is Simpson’s evenness (left, [N = 256, P = 0.75) and clonality (right, [N = 256, P = 0.69]) and diversity that is Shannon entropy (left, [N = 256, P = 0.22]), and Simpson’s diversity (right, [N = 256, P = 0.25]) in blood collected from patients at the time of primary resectable disease or collected during metastatic disease. **D-I)** P values one-way ANOVA or from Kruskal–Wallis H-test for plots with dashed lines. **A-B, D-I)** black bars represent the means, or dashed line represent median and interquartile range. N=number of patients. Source data

We compared T cell repertoires between all primary and metastatic tumors and found no difference in templates per ng or Shannon entropy, but primary tumors had more productive rearrangements, lower Simpson’s evenness and lower Simpson’s diversity, consistent with higher clonality (Fig. 5i and Extended Data Fig. 7m). We also found that primary tumors had a higher fraction of the tumor TCR repertoire occupied by clones with more than one template on average per patient than metastatic tumors (that is expanded clones; Fig. 5j). Although metastases in general had reduced clonal TCR repertoires (Fig. 5i), the low pORG and lung cohort metastases had TCR repertoires indicating increased clonal responses (low evenness/high clonality) relative to high pORG and liver cohort, respectively (Fig. 5e,k), consistent with their better prognosis. We also parsed out specific metastatic collection sites in the TCR-seq dataset which showed the composition of high and low pORG metastases and liver and lung cohort metastases (Extended Data Figs. 7n and 8a). Grouping by metastatic collection site demonstrated lung metastases have greater clonality than liver metastases, consistent with our cohort-level data (Extended Data Fig. 8a,b).

### Higher peripheral TCR clonality with low pORG metastases

**Extended Data Fig. 9:**
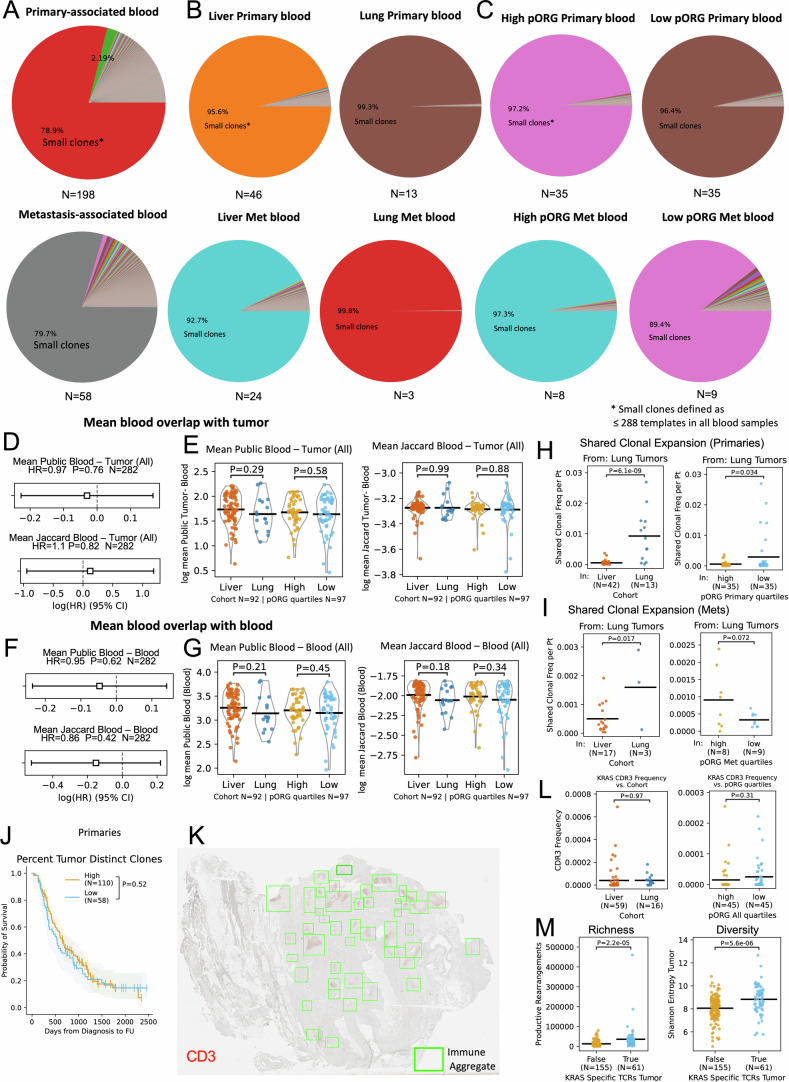
Association of organotropism or KRAS-specific TCRs with clonal expansion. **A-C)** Pie charts of fraction of each cohorts’ repertoire made up of expanded clones, where the largest slice is all the small clones, that is those present in less than or equal to one template per patient on average in the blood and each additional slice of pie is an expanded clone present at greater than 1 template per patient on average across the blood samples. Comparisons are as follows: **A)** Blood from patients with primary-associated (N = 198 pts.) or metastatic-associated (N = 58 pts.) disease, **B)** Blood from patients in the liver (primary [N = 46 pts.], metastatic [N = 24 pts.]) or lung cohort with primary (N = 13 pts.) or metastatic (N = 3 pts.) disease, **C)** Blood from patients in the high (primary [N = 35 pts.], metastatic [N = 8 pts.]) or low pORG cohort with primary (N = 35 pts.) or metastatic (N = 9 pts.) disease. **D)** Cox proportional hazards (CPH) modeling of overall survival (OS) for mean public overlap (top, [N = 282, P = 0.76]) and Jaccard index (bottom, [N = 282, P = 0.82]) of blood samples with each tumor’s clonotypes. **E)** Mean public overlap (left, liver or lung [N = 92, P = 0.29], high or low pORG quartile [N = 97, P = 0.58]) and Jaccard index (right, liver or lung [N = 92, P = 0.99], high or low pORG quartile [N = 97, P = 0.88]) of blood samples with each tumor’s clonotypes in liver versus lung and high versus low pORG cohorts. **F)** CPH modeling of OS versus mean public clones (top, [N = 282, P = 0.62]) and Jaccard overlap (bottom, [N = 282, P = 0.42]) of blood samples with each blood sample’s clonotypes. **G)** Mean public overlap (left, liver or lung [N = 92, P = 0.21], high or low pORG quartile [N = 97, P = 0.45]) and Jaccard index (right, liver or lung [N = 92, P = 0.18], high or low pORG quartile [N = 97, P = 0.34]) of blood samples with each blood sample’s clonotypes of cohorts as in (**E**). **H)** CDR3 frequency of all shared clonal sequences from lung present in each patient’s primary tumor repertoire in liver (N = 42) versus lung (N = 13, P = 6.1e-09) cohort (left) and high (N = 3) versus low (N = 35, P = 0.034) pORG primaries (right). **I)** CDR3 frequency of all shared clonal clonotypes from lung present in each patient’s metastatic tumor repertoire in liver (N = 17) versus lung (N = 3, P = 0.017) cohort (left) and high (N = 8) versus low (N = 9, P = 0.072) pORG metastases (right). **J)** Kaplan–Meier estimate of OS for patients with high (N = 110) versus low (N = 58, P = 0.52) tumor-distinct clones in primaries. P values between groups indicated with brackets determined by log-rank test and shaded regions represent 95% confidence intervals. **K)** Example of identification of lymphoid aggregates in mIHC data; Defined as CD20+ cells clustered with CD3+ cells present. N = 12 tissues analyzed. **L)** CDR3 frequency of putative mutant KRAS-specific TCRβ sequences in tumor samples in liver (N = 59) versus lung (N = 16, P = 0.97) cohort (left) and the top (N = 45) versus bottom (N = 45, P = 0.31) quartile of pORG tumors by GSVA scores from all patients (right). **M)** TCRβ repertoire richness (left, true [N = 61], false [N = 155, P = 2.2e-05]) and diversity (right, true [N = 61]. False [N = 155, P = 5.6e-06]) in tumors with and without putative mutant KRAS-specific sequences in tumor. **D, F, J)** Patients who died within 30 days after resection are not shown. N=number of patients. **E-G)** P values from one-way ANOVA, black bars represent the means. N=number of patients. **H-M)** P values from two-tailed t-test, black bars represent the means. N=number of patients. Source data

Peripheral blood TCR clonality at baseline and expansion of clones post-treatment was reported to predict survival in patients with metastatic PDAC treated with ICIs^39^. In our dataset, we found trends for longer survival in patients with high blood TCR clonality and lower TCR diversity metrics (Extended Data Fig. 8c); and elevated TCR clonality/lower diversity in blood from patients with low pORG versus high pORG metastatic disease (Fig. 5l and Extended Data Fig. 8d), but this difference was not seen in blood collected at or before primary tumor resection (Extended Data Fig. 8d–f). In contrast, blood samples from lung cohort patients had greater Simpson’s evenness and a trend toward lower clonality (Extended Data Fig. 8e,g). Blood TCR richness showed no difference between high and low pORG or liver and lung cohorts (Extended Data Fig. 8h). Comparison of all blood samples associated with primary versus metastatic disease did not identify significant differences in TCR repertoire richness, evenness or diversity (Extended Data Fig. 8i) or the number of expanded clones (Extended Data Fig. 9a). Compared to the liver cohort, lung cohort primary-associated blood samples had fewer expanded clones (Extended Data Fig. 9b), consistent with their significantly higher blood TCR evenness, while low pORG relative to high pORG blood TCR repertoires displayed higher fractions of expanded clones in metastatic disease (Extended Data Fig. 9c).

### Shared TCR clonal responses in low pORG, lung cohort tumors

**Fig. 6:**
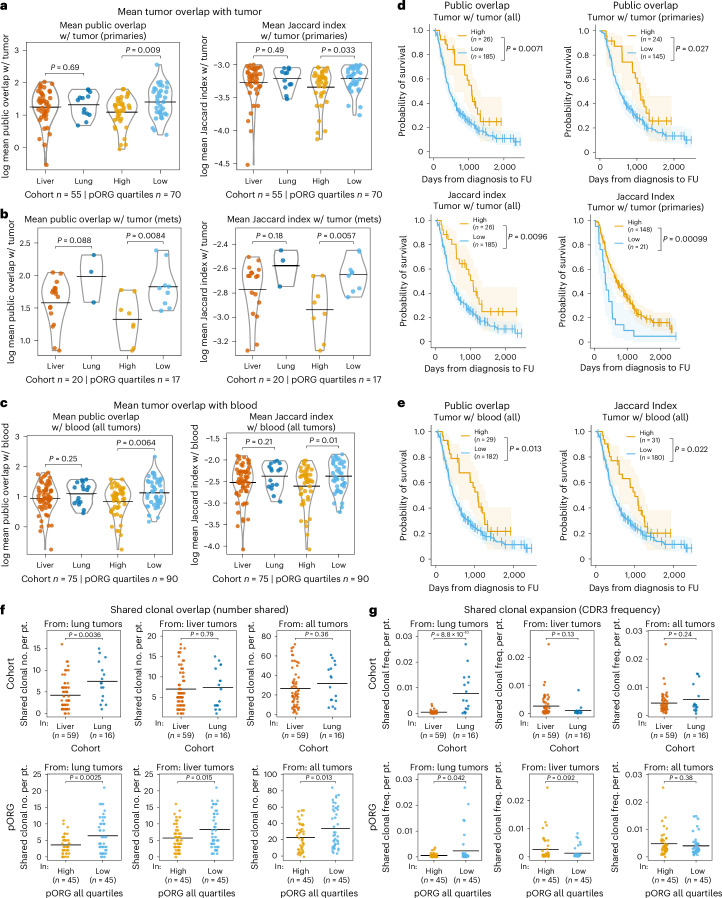
Shared, clonal TCR responses in low pORG, lung cohort tumors. **a**,**b**, Number and fraction of TCRβ clonotypes that are shared with other tumor clonotypes in the dataset, quantified as (log) mean public overlap and (log) mean Jaccard index (intersection of two sets over the union of two sets), respectively. Primary tumors’ mean public overlap (left; liver or lung (*n* = 55 pts., *P* = 0.69), pORG quartile high or low (*n* = 70 pts., p = 0.009)) and Jaccard index (right; liver or lung (*n* = 55 pts., *P* = 0.49), high or low (*n* = 70 pts., *P* = 0.033)) of each tumor with each other tumor sample, grouped by the indicated cohorts (**a**). Metastatic tumors’ (mets) mean public overlap (left; liver or lung (*n* = 20 pts., *P* = 0.088), pORG quartile high or low (*n* = 17 pts., *P* = 0.0084)) and Jaccard index (right; liver or lung (*n* = 20 pts., *P* = 0.18), pORG quartile high or low (*n* = 17 pts., *P* = 0.0057)), grouped by the indicated cohorts (**b**). **c**, Mean public overlap (left; liver or lung (*n* = 75 pts., *P* = 0.25), high or low (*n* = 90 pts., *P* = 0.0064)) and Jaccard indices (right; liver or lung (*n* = 75 pts., *P* = 0.21), high or low (*n* = 90 pts., *P* = 0.01)) of tumors’ overlap with each blood sample, grouped by the indicated cohorts. **d**, K–M estimates of OS of patients with high versus low mean public clonotypes (top) of all tumor samples’ (left; high (*n* = 26 pts.) or low (*n* = 185 pts.), *P* = 0.0071) or primary tumor samples’ (right; high (*n* = 24 pts.) or low (*n* = 145 pts.), *P* = 0.027) and Jaccard index (bottom) of all tumor samples’ (left; high (*n* = 26 pts.) or low (*n* = 185 pts.), *P* = 0.0096) or primary tumor samples’ (right; high (*n* = 148 pts.) or low (*n* = 21 pts.), *P* = 0.00099) overlap with tumor TCRβ repertoires. **e**, K–M estimates of OS of patients with high versus low mean public overlap (left; high (*n* = 29 pts.) or low (*n* = 182 pts.), *P* = 0.013) and Jaccard index (right; high (*n* = 31 pts.) or low (*n* = 180 pts.), *P* = 0.022) of all tumor samples’ overlap with blood repertoires. **f**, Number of shared, dominantly clonal CDR3 clonotypes from lung (left; liver (*n* = 59), lung (*n* = 16), *P* = 0.036), liver (center; liver (*n* = 59), lung (*n* = 16), *P* = 0.79) and all tumors (right; liver (*n* = 59), lung (*n* = 16), *P* = 0.36) present in each patient’s repertoire in liver versus lung cohort (top row). Number of shared, dominantly clonal CDR3 clonotypes from lung (left; high (*n* = 45), low (*n* = 45), *P* = 0.0025), liver (center; high (*n* = 45), low (*n* = 45), *P* = 0.015) and all tumors (right; high (*n* = 45), low (*n* = 45), *P* = 0.013) present in each patient’s repertoire in high versus low pORG quartiles (bottom row). **g**, Productive frequency of all shared, dominantly clonal CDR3 clonotypes from lung (left; liver (*n* = 59), lung (*n* = 16), *P* = 8.8 × 10^−10^), liver (center; liver (*n* = 59), lung (*n* = 16), *P* = 0.13) and all tumors (right; liver (*n* = 59), lung (*n* = 16), *P* = 0.24) present in each patient’s repertoire in liver versus lung cohort (top row). Productive frequency of all shared, dominantly clonal CDR3 clonotypes from lung (left; high (*n* = 45), low (*n* = 45), *P* = 0.042), liver (center; high (*n* = 45), low (*n* = 45), *P* = 0.092) and all tumors (right; high (*n* = 45), low (*n* = 45), *P* = 0.38) present in each patient’s repertoire in high versus low pORG (bottom row). *P* values were obtained by one-way ANOVA; black bars represent the mean (**a**–**c**). High/low cutoff determined with the ROC and maximum Youden’s index; *P* values were determined by a log-rank test and shaded regions represent 95% CI (**d**,**e**). Patients who died within 30 days after resection are not shown. *P* values are from a two-tailed *t*-test, black bars represent the mean, and *n* indicates the number of patients (**f**,**g**). Source data

To assess responses to potential common antigens, we evaluated overlap in the tumor and blood TCR repertoires in our cohorts using two metrics, public overlap and Jaccard index^37^. Public overlap counts the number of clonotypes shared between two groups. Jaccard index is defined as the size of the intersection over the size of the union of two sample sets. We calculated the mean of each sample’s public overlap and Jaccard index with every other sample’s TCR repertoire, indicative of a sample’s T cell response to common antigens. We found greater overlap between low pORG tumors and other tumors (Fig. 6a,b) or blood samples (Fig. 6c) and a correlation between these overlaps and survival for all patients and primary-sampled patients (Fig. 6d,e). In contrast, blood repertoires overlap with either tumor or other blood repertoires did not show associations with survival, liver versus lung cohort or high versus low pORG tumors (Extended Data Fig. 9d–g). These data demonstrate that an increased proportion of shared clonotypes found in the tumor are associated with favorable disease biology (lower pORG scores) and better clinical outcomes in PDAC.

For cohort-specific investigation of clonal TCRs in each group (liver, lung or all tumors), we identified CDR3 sequences that were shared by at least 25% of samples in a group and were dominantly clonal in at least one sample, hereafter referred to as ‘shared clonal’. Low pORG tumors had greater numbers of shared clonal sequences from any of the groups, liver, lung or all, than high pORG tumors, whereas lung cohort tumors trended this way but had significantly more shared clonal sequences from the lung cohort (Fig. 6f), suggesting unique lung cohort T cell responses. The frequency of the shared clonal sequences in each patient’s tumor revealed that in lung cohort and low pORG tumors, lung cohort shared clonal sequences were expanded to a larger proportion of the repertoire (Fig. 6g), and this was true separately in lung cohort primaries and metastases, and low pORG primaries but not metastases (Extended Data Fig. 9h,i). These results suggest that lung cohort patients may be a subset of low pORG tumor patients who harbor unique shared TCRβ clonal sequences that undergo a selective expansion.

### Clonal expansion within tumors associated with better outcome

**Fig. 7:**
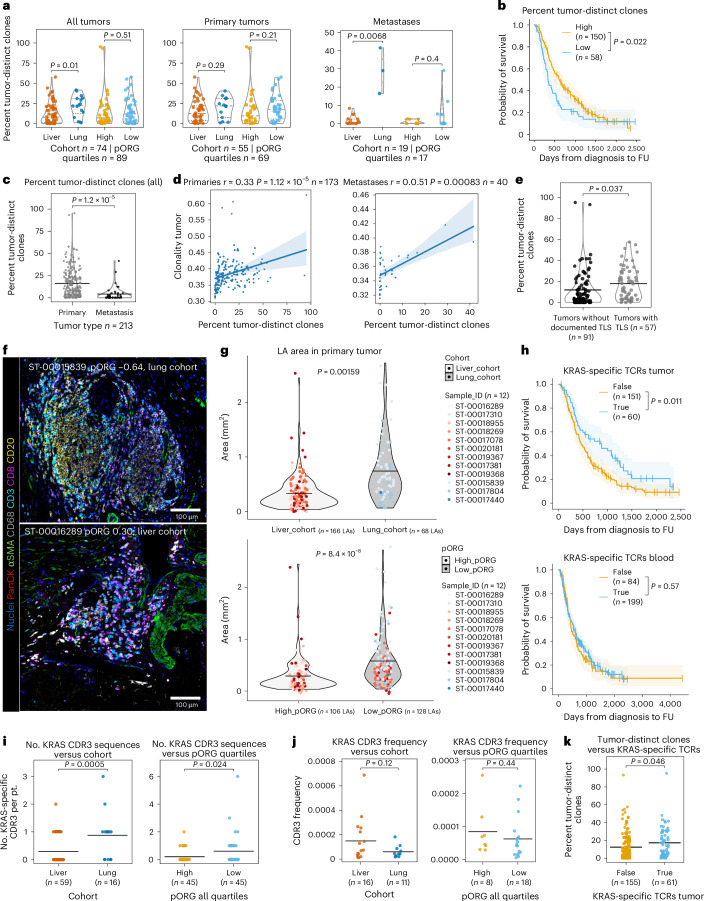
T cell clonal expansion within tumors associated with better outcome. **a**, The percentage of unique tumor TCRβ CDR3 sequences with ≥10 templates detected in tumor samples, but not in patient matched blood samples; all tumors (left; liver or lung (*n* = 74 pts., *P* = 0.01), pORG quartile high or low (*n* = 89 pts., *P* = 0.51)), primary tumors (center; liver or lung (*n* = 55 pts., *P* = 0.29), high or low (*n* = 69 pts., *P* = 0.21)) and metastatic tumors (right; liver or lung (*n* = 19 pts., *P* = 0.0068), high or low (*n* = 17 pts., *P* = 0.4)) from the indicated cohorts. *P* values from Kruskal–Wallis H-test; dashed lines represent median and IQR. **b**, K–M estimates of OS of patients with high (*n* = 150 pts.) versus low (*n* = 58 pts.; *P* = 0.022) tumor-distinct clones. High/low cutoff determined with the ROC and maximum Youden’s index. **c**, Percent tumor-distinct clones in primary tumors versus metastases (*n* = 213 pts., *P* = 1.2 × 10^−5^). *P* values were derived from a from one-way ANOVA. **d**, Correlation between tumor-distinct clones and tumor TCRβ clonality quantified by 1 − normalized Shannon entropy for primaries (left; *r* and *P* value from Pearson correlation (*n* = 173 pts., *P* = 1.12 × 10^−5^)) and metastases (right; *r* and *P* value from Spearman correlation (*n* = 40 pts., *P* = 0.00083)). The line represents a linear regression and shaded regions show the 95% CI. **e**, The percentage of tumor-distinct clones in tumors with the presence (*n* = 57 pts.) or absence (*n* = 91 pts.; *P* = 0.037) of pathologist-identified TLSs. **f**, Representative mIHC images of LAs in lung/low pORG (top) and liver/high pORG (bottom) primary tumors; *n* = 12 images collected. **g**, LA area in primary tumors from patients in the liver cohort (nine patients (*n* = 166 LAs evaluated)) versus lung cohort (three patients (*n* = 68 LAs; *P* = 0.00159)) (top) or high pORG (seven patients (*n* = 106 LAs)) versus low pORG (five patients (*n* = 128 LAs; *P* = 8.4 × 10^−8^)) (bottom). Each point represents one immune aggregate colored by patient specimen. **h**, K–M estimates of OS of patients containing at least one putative mutant KRAS-specific TCRβ sequence within their TCR repertoire in tumors (top; present (*n* = 60 pts.), not detected (*n* = 151 pts.), *P* = 0.011) or blood (bottom; present (*n* = 199 pts.), not detected (*n* = 84 pts.), *P* = 0.57) for all patients. **i**, Number of putative mutant KRAS-specific TCRβ sequences within the TCR repertoire of each tumor (primary and metastasis) in liver versus lung cohort (left; liver (*n* = 59 pts.), lung (*n* = 16 pts.), *P* = 0.0005) and the top versus bottom quartile of pORG tumors by GSVA scores from all tumors (right; high (*n* = 45 pts.), low (*n* = 45 pts.), *P* = 0.024). **j**, CDR3 frequency of putative mutant KRAS-specific TCRβ sequences in samples containing them in liver versus lung cohort (left; liver (*n* = 16 pts.), lung (*n* = 11 pts.), *P* = 0.12) and the top versus bottom quartile of pORG tumors by GSVA scores from all patients (right; high (*n* = 8 pts.), low (*n* = 18 pts.), *P* = 0.44). **k**, Percent tumor-distinct clones in tumors with (*n* = 61 pts.) or without (*n* = 155 pts.; *P* = 0.046) putative KRAS-specific TCRβ sequences in the tumor. Black bars represent the mean (**c**,**e**,**g**,**i**–**k**). *P* values from two-tailed *t*-test (**e**,**g**,**i**–**k**). *P* values were determined by a log-rank test and shaded regions represent 95% CI (**b**,**h**). Patients who died within 30 days after resection are not shown. Source data

We considered that expanded T cell clones occurring in tumors but not sampled in the blood TCR repertoire may reflect new clonal development in tumors. We found that lung cohort tumors harbored significantly more of these tumor-distinct clones than liver cohort tumors, especially in metastases (Fig. 7a). Higher tumor-distinct clones were associated with better OS in all patients, but not in patients with primary tumor resections alone (Fig. 7b and Extended Data Fig. 9j). Primary samples had more tumor-distinct clones than metastases (Fig. 7c). Additionally, clonality positively correlated with the percentage of tumor-distinct clones in primary tumors and metastases (Fig. 7d), underscoring that new clonal development may contribute to overall clonality of the tumor T cell repertoire.

Clonal T cell responses to tumor-associated antigens may arise in TLSs^42^. Consistent with this, we found a significantly higher percentage of tumor-distinct clones in tumors that were characterized by two blinded, board-certified pathologists (T.M. and B.B.) as containing at least one TLS (Fig. 7e). Furthermore, lung cohort metastases, which have significantly more tumor-distinct clones, had more TLSs than liver cohort metastases (Extended Data Fig. 2b). Moreover, we identified LAs of CD20^+^ cells clustered with CD3^+^ cells in mIHC images from the nine liver and three lung cohort primary tumor sections analyzed in Fig. 4 (Fig. 7f and Extended Data Fig. 9k). Although there were no significant differences in the average number of LAs between liver and lung cohorts, LAs from lung cohort primaries were on average twice the size of those from liver cohort, and this was also true when specimens were divided into low versus high pORG tumors (Fig. 7g).

To investigate T cell responses to PDAC-initiating antigens, we assessed 21 published CDR3 sequences experimentally confirmed to be part of TCRβ receptors specific for KRAS G12/13 alterations^43–46^ that commonly drive PDAC tumors (though we were unable to confirm the presence of the reported matching HLA allele). The presence of these putative mutant KRAS-specific CDR3 sequences in tumors from all patients was associated with better patient outcome, but this was not the case for their presence in blood repertoires (Fig. 7h). We identified higher numbers of KRAS-specific sequences present per patient in lung cohort and low pORG tumors (Fig. 7i) but not significantly higher productive frequency of these sequences in either liver versus lung or high versus low pORG cohort comparisons (Fig. 7j and Extended Data Fig. 9l). Comparison to additional metrics revealed that tumors with putative mutant KRAS-specific clones present had increased TCRβ tumor repertoire richness, diversity and tumor-distinct clones (Fig. 7k and Extended Data Fig. 9m). However, only two of over 21,000 tumor-distinct clones identified and none of the shared, clonally dominant sequences in our cohorts (Fig. 6f,g) matched those reported to be mutant KRAS specific. Together, these results suggest that the presence of T cells reactive to tumor-initiating, persistent neoepitopes, like mutant KRAS, in tumors may associate with better patient outcome and liver-adverse metastatic disease, but selective clonal expansion in lung cohort or low pORG tumors associated with their better outcome does not often involve expansion of these clones; however, further discovery of additional putative mutant KRAS CDR3s and HLA tumor-matching is required to validate these hypotheses.

## Discussion

Previous efforts to divide PDAC tumors into subtypes used unbiased approaches to describe mutually exclusive subsets^9,11,13,14,47,48^. We took an alternative approach of classifying tumors based on the observed association between metastatic organotropism and better clinical outcomes in patients with lung-avid/liver-averse disease^1–3^. As previously reported^16^, tumors from patients in the lung cohort were unlikely to be categorized as the basal-like subtype, whereas classical subtype tumors were common in both liver and lung cohorts. Uniquely, we found that patients with classical subtype primary tumors fared significantly worse if their disease was liver-avid rather than lung-avid/liver-averse.

We extracted a set of overexpressed genes from liver-avid primary tumors that were not DE in basal-like versus classical primary tumors (pORG). While gene expression differences between liver versus lung cohort primary tumors were relatively weak compared to basal-like versus classical, and the accuracy of the pORG gene set for predicting recurrence site will require validation in outside cohorts, we demonstrated that this pORG gene set can independently predict patient outcomes. Furthermore, high pORG and liver-avid primary tumors were both enriched for cell cycle, replication and DNA repair pathways, indicative of ongoing RS tolerance. Accordingly, RS foci in tumor cells and specifically in Ki67^+^ proliferating tumor cells were more abundant in tumors with high pORG scores. Liver-avid tumors with somatic alterations in DDR genes had some of the highest pORG scores, suggesting that PDAC tumor cells can avoid the detrimental effects of ongoing DNA damage by adopting strong RS response mechanisms. Conversely, OS was better in patients with tumors that have low pORG scores, particularly if they harbor a DDR gene mutation, likely due to failure to adapt to RS caused by a defective DNA repair network and suggesting that low pORG tumors are less fit and may be more sensitive to therapeutics that interrupt DDR pathways. A high pORG signature was also associated with an IRDS^30–32^, which unlike an acute type 1 IFN response, can reduce tumor immunity^33^. Multiplex imaging of immune phenotypes supported this hypothesis by demonstrating that low pORG primary and lung cohort primary tumors both had increased B and T cells with decreased myeloid subsets; and this was further supported by deconvolution of the bulk RNA-seq to estimate immune cell types. This increase in tumor immunity is consistent with an inability to tolerate genomic instability and RS associated DNA damage in low pORG tumors.

We extended these immune observations with TCRβ sequencing. In relation to tumor immunity, both diverse TCRβ repertoires as well as clonal expansion are reported to associate with positive outcomes in patients with PDAC^20,39^. Consistent with these reports, we identified high TCR richness and diversity in low pORG tumors and this was associated with better patient outcomes. Increased TCR diversity was also associated with increased shared/public clonotypes, which were also associated with low pORG tumors and longer OS, suggesting common tumor-controlling immune responses in low pORG tumors. Additionally, we found that high tumor clonality/low evenness was prognostic for longer OS, and clonality was significantly increased in lung and low pORG metastases relative to liver and high pORG metastases, in contrast to the trend observed in metastases in general, which showed reduced clonality compared to primary tumors. We found that the increased tumor clonality was associated with increased T cell clones found in tumors that were absent in paired blood samples; suggesting new clonal expansion of T cells that are not yet detected in blood. These tumor-distinct clones were higher in the lung cohort compared to the liver cohort tumors and associated with the presence of TLSs. Possible explanations for these observations are that lung cohort patients may have unique mechanisms for T cell clonal development and/or that only patients who stochastically develop new T cell responses directed toward the correct antigens end up with liver-averse disease.

As in all clinical studies of this nature, we acknowledge that limited follow-up time and confounding variables provide possible limitations to our study. In vivo experiments, combined with additional clinically annotated patient datasets, are needed to further validate hypotheses regarding metastatic seeding and/or survival of these PDAC subtypes. Preclinical follow-up could reveal additional mechanistic insights as well as biomarkers for avenues of therapeutic intervention in either the neoadjuvant and/or adjuvant settings.

## Methods

### Tissue acquisition and patient consent

Our research complies with all relevant ethical regulations and was approved under Oregon Health and Science University (OHSU) Institutional Review Board protocol no. 00003609. Patient data, blood and tissues were obtained with informed consent in accordance with the Declaration of Helsinki and were acquired through the Oregon Pancreas Tissue Registry. Patients were not compensated for participation.

### Clinical data collection

From a de-identified dataset of 1,873 patients diagnosed with and/or treated for PDAC at our institution between 2004 and 2020, we identified 422 patients for which we had specimens with sequencing data (*n* = 374) and/or specific evidence of disease metastasis site(s) from the OHSU cancer registry and disease-relevant CT scans to allow cohort classification. Patients whose primary tumor was located at the ampulla of Vater but classified as pancreatobiliary subtype were included (*n* = 9). Clinical course time points, stage, grade, nodal involvement, resection margins and angiolymphatic invasion were provided as de-identified data by the OHSU cancer registry with quality control data verification by pathologists (B.B. and T.M.). Patient demographics were also collected and include age and self-reported sex. We reviewed all available CT scans for all patients with primary tumor resection dates recorded by the cancer registrar, with tumor samples analyzed by RNA-seq, DNA-seq or TCR-seq, and/or with additional information indicating metastatic spread (for example, metastatic samples received for related studies). We abstracted the site of all lesions proven to be metastatic by biopsy and/or that clearly increased in size during progression or decreased in size during treatment as long as a radiologist described the lesion as ‘likely’, ‘suspicious for’, ‘concerning for’ or ‘favor’ metastasis. Clinical imaging was reviewed by a radiologist (A.G.) to validate patient assignments to the liver, lung and neither liver nor lung (other recurrence site) cohorts. To adhere to our clinical definition, we did not exclude patients from any cohort due to short survival. Time to recurrence after surgical removal of tumor and disease-free status was calculated from the earliest of either the recurrence date provided by the OHSU cancer registry, or the date of earliest lesion abstracted from CT reports. All patient information was frozen in July 2021.

### Specimen processing

Primary and metastatic PDAC tumor specimens from consented patients at OHSU were processed by the OHSU Department of Pathology and preserved by standard FFPE. FFPE sections of 3–4 μm were stained with hematoxylin and eosin (H&E) and used for other protein staining procedures.

### Histology data

H&E-stained FFPE tissue sections from regions corresponding to those extracted for RNA-seq and somatic alteration analyses were independently appraised by two pathologists (B.B. and T.M.) blinded to study cohorts for the histologic features shown in Table 1.

### Tempus RNA-seq and genomic alteration panel processing

OHSU provided FFPE PDAC specimen blocks along with matched normal blood or tissue to Tempus as part of a contract agreement. OHSU pathologist (T.M.) and Tempus pathologists marked regions of high tumor content (>20% ratio of tumor to normal nuclei) on H&E-stained slides for DNA and RNA extraction. Solid tumor total nucleic acid was extracted from these tumor regions on adjacent FFPE tissue sections using Chemagic 360 sample-specific extraction kits (PerkinElmer, cat. no. 41581) and digested by proteinase K (Thermo Fisher, cat. no. EO0492). RNA was purified from the total nucleic acid by DNase-I digestion (Thermo Fisher, cat. no. 89836). DNA sequencing of 596 genes and whole-transcriptome RNA sequencing were performed as described^49,50^. Briefly, 100 ng of DNA for each tumor sample was mechanically sheared to an average size of 200 bp using a Covaris Ultrasonicator. DNA libraries were prepared using the KAPA Hyper Prep kit (Roche, cat. no. KRO961), hybridized to the xT probe set and amplified with the KAPA HiFi HotStart ReadyMix (Roche, cat. no. KK2602). One hundred ng of RNA for each tumor sample was heat fragmented in the presence of magnesium to an average size of 200 bp. Library preps were hybridized to the xGEN Exome Research Panel v.1.0 (Integrated DNA Technologies, cat. no. 10005153) and target recovery was performed using streptavidin-coated beads, followed by amplification with the KAPA HiFi Library Amplification kit (Roche, cat. no. KK2612). The amplified target-captured DNA tumor library was sequenced using 2 × 126-bp paired-end reads to an average unique on-target depth of 500× (tumor) and 150× (normal) on an Illumina HiSeq 4000. The amplified target-captured RNA tumor library was sequenced using 2 × 75 bp paired-end reads to an average of 50 million reads on an Illumina HiSeq 4000. Samples were further assessed for uniformity with each sample required to have 95% of all targeted bp sequenced to a minimum depth of 300×. Raw fastq files were returned to OHSU as well as PDF reports of summarized DNA alterations.

### DNA sequence analysis

DNA variant detection, reporting and copy number analysis were performed as described^50^. Alignment and mapping were to GRCh37 using Novo align + BWA. Copy number variants were derived from proprietary tumor–normal match analysis using CNAtools. Matched normal DNA was available for most tumor specimens and if not available, a pool of normal samples was used to call variants. For cases relying on a pooled normal, there is an increased risk of true germline mutations being identified as somatic^49^. Genomic variants and annotations are displayed on oncoprints using the Oncoprint function from the Complex Heatmap R package^51^. Cohort and survival analysis were performed as follows on alterations present in more than nine patients. Fisher’s exact tests were used to determine whether the alteration prevalence differed significantly between cohorts; FDR correction was performed with the Benjamini–Hochberg method. We determined whether each gene alteration (annotated as gain of function or loss of function or simply ‘altered’), may influence patient survival and found that only *ARID1A* variants had annotation type-dependent prognostic value (with ARID1A loss of function conferring better prognosis relative to wild-type (WT) and *ARID1A* altered); therefore, we pooled alteration types for single-variable Cox proportional hazards modeling of gene alterations versus OS, with the exception of *ARID1A*.

### RNA sequencing analysis

Paired-end fastq sequences were trimmed using Trim Galore (v.0.6.3) and default parameters. Pseudoalignment was performed with kallisto (v.0.44.0) using genome assembly GRCh38.p5 and GENCODE (v.24) annotation; default parameters were used other than the number of threads. The Bioconda package bioconductor-tximport (v.1.12.1) was used to create gene-level counts and abundances (TPMs). Quality checks were assessed with FastQC (v.0.11.8) and MultiQC (v.1.7). Quality checks, read trimming, pseudoalignment and quantitation were performed via a reproducible snakemake pipeline, and all dependencies for these steps were deployed within the anaconda package management system^52,53^.

### PurIST analysis

PurIST subtype calls and scores were generated using the PurIST method^12^ applied to our RNA-seq data. The PurIST authors provide instructions, R scripts and gene pairs on GitHub (https://github.com/naimurashid/PurIST).

### Development of pORG and pSUB gene sets

A two-factor analysis with DESeq2 (ref. ^22^) was performed on RNA-seq counts from the 76 primary samples in the liver and lung cohorts after filtering out low expressing genes using a TPM cutoff of <0.25 average expression across the dataset. The two factors modeled were: primary tumor liver cohort versus lung cohort and basal-like versus classical (from PurIST subtyping). The signal for the clinical liver versus lung factor was weaker than that for the RNA-based subtype factor. To select appropriately sized gene sets for GSVA, we chose a permissive FDR-adjusted *P* value cutoff for individual genes of 0.2 for the liver versus lung factor and a restrictive cutoff of 0.0001 for the basal-like versus classical factor. For pORG, we selected DE genes from the liver versus lung factor (FDR < 0.2), then excluded genes that co-occurred in the top half of the ranked genes from the basal versus classical factor, resulting in a list of 55 upregulated genes (only genes up in liver cohort were selected). For pSUB, we selected DE genes up for the basal-like versus classical factor (FDR < 0.0001), then excluded genes that co-occurred in the top half of the ranked genes from the liver versus lung factor, resulting in a list of 51 upregulated genes. To test for over-fitting, we performed a leave-one-out cross validation by repeating the two-factor modeling steps above with each of the 76 samples left out one at a time. For each iteration, the resulting gene set was used to calculate GSVA scores on all primary samples. Once all iterations were complete, the GSVA scores from the left-out sample from each iteration were combined to generate a cross-validated GSVA matrix. The cross-validated GSVA scores for pORG still correlated as expected with the liver versus lung labels (*P* = 0.033), but not as well as the over-fit scores did. Likewise, leave-one-out cross-validated GSVA scores were calculated and tested for pSUB. pSUB scores correlated as expected with the basal-like versus classical labels (*P* = 3.1 × 10^−9^) and did not show much over-fitting bias.

### GSEA and GSVA analyses

The GSVA tool^23^ was used with log scaled, TMM-normalized CPM data^54^ to calculate relative pORG and pSUB gene set scores across all primaries, all metastases and all tumors and identify top/bottom quartile cohorts. GSEA^55^ was run on clinical liver and lung cohorts, pORG, pSUB and PurIST top/bottom quartile cohorts using the MSigDB database v.7.5.1 Hallmark gene set collection^26^. The eight genes used for the IRDS signature were: *STAT1*, *IFI44*, *IFIT3*, *OAS1*, *IFIT1*, *ISG15*, *MX1* and *USP18*. To calculate GSVA scores for Hallmarks and other signatures, the DESeq2 R library was used to import raw RNA-seq data via txi import and perform variance stabilized transformation for downstream GSVA analysis. The GSVA R library was then used to calculate GSVA scores for gene sets, including the Hallmark gene set collection from the MSigDB database (v.7.5.1), response to IFN gene sets GO:0034341 and GO:0071357 from org.Hs.eg.db (v.3.17.0) and T and B cell signatures for profiling the TME^56^. To produce heatmaps of primary tumor GSVA results, we used the R package pheatmap (v.1.0.12)^57^. Tumor samples (columns) were ordered from highest to lowest pORG or pSUB, whereas MSigDB Hallmarks (rows) were hierarchically clustered using default pheatmap function parameters. Samples from patients who were not resected nor in the liver/lung cohort were excluded (*n* = 6). Before running the pheatmap function, GSVA results were subset to include only MSigDB Hallmarks that were significantly different by GSEA (FDR *P* adjusted < 0.05) for high/low pORG or pSUB groups, respectively.

### Single-cell RNA-seq analysis of public data

For single-cell analysis of pORG and pSUB gene sets, we obtained single-cell RNA-seq profiles of primary PDAC tumors and liver metastases from the National Institutes of Health (NIH) Gene Expression Omnibus (GEO) (GSE205013)^25^. Primary tumors and liver metastases were analyzed separately using the R package Seurat (v.4.3.0)^58^ but run through the same computational workflow. Per-cell quality cutoffs were set to the same parameters originally used by Werba et al.^25^ at 1,500 min; reads, 500 min; unique genes detected; read percentage from mitochondrial genes <15%; and read percentage <1% from erythroid genes (*ALAS2*, *HBA1*, *HBA2*, *HBB* and *HBM*). For data integration, we applied Seurat’s SCTransform^59^ and RPCA integration workflow. Briefly, we applied the SCTransform function with ‘v2’ regularization to each sample, selected the top 3,000 features for integration through SelectIntegrationFeatures and ran principal-component analysis on each sample via RunPCA. Integration was performed using FindIntegrationAnchors with normalization set to ‘SCT’ and reduction set to ‘rpca’, followed by IntegrateData with normalization method set to ‘SCT’. After integration, Uniform Manifold Approximation and Projection (UMAP) was performed on principal components 1:30, and clustering was run with FindClusters resolution set to 0.7. Finally, RNA count data were then normalized and scaled for all downstream analysis. To identify cell types, we ran the FindAllMarkers function to find highly expressed genes in each cluster. We labeled clusters by cell type in accordance with the cell-type markers used by Werba et al.^25^, with the exception of a hepatocyte cluster identified by high albumin (*ALB*) gene expression found in the liver metastasis data. Clusters representing epithelial–endothelial doublets from the primary tumors and epithelial–myeloid doublets from the liver metastases were identified from high coexpression of cell type markers and consequently removed. Following cell type identification, we computed module scores for the pORG and pSUB gene sets on a per-cell basis using the function AddModuleScore with default settings.

### VIPER analysis and immune cell type estimation

The transcriptional regulon enrichment analysis was performed using VIPER with the TCGA PAAD ARACNe-inferred network^27,28^. Gene expression data were normalized before running VIPER by median centering and scaling. VIPER regulon scores for all primaries were used for cohort comparisons. Immune cell type estimation was run using the R package immunedeconv (v.2.1.0)^60^ and selecting the quantiseq^61^, mcp_counter^62^, xCell^63^ and epic^64^ algorithms. To perform Gene Ontology (GO)^65,66^ enrichment analysis for regulons increased in high pORG samples and liver cohort samples, we used the R package ClusterProfiler (v.4.6.2)^67^. The ClusterProfiler function enrichGO was set to test GO biological process terms, threshold results at 0.05 *P* and *q*-value, and use all regulons as the background. Jaccard similarity was calculated through the function pairwise_termsim with default settings. Enrichment maps were plotted with the R package enrichplot (v.1.18.4)^68^.

### Immunofluorescence multiplex imaging

A PDAC TMA was constructed at OHSU using FFPE blocks from tumors analyzed by RNA-seq and included 1–2 cores each from 34 primary tumors (55 cores in total). Immunofluorescence staining, imaging and image processing were performed on the TMA as described^69^. Briefly, images were scanned with the Zeiss Axioscan Z1, acquired, stitched and exported to tiff format using Zeiss Zen Blue software (v.2.3), registered using MATLAB (v.9.11.0), followed by cellular segmentation using Cellpose^70^ or Mesmer^71^ algorithms. Unsupervised clustering of single-cell mean intensity was used to define cell types, using the Leiden algorithm implemented in scanpy (v.1.9.3)^72^. Ki67^+^ epithelial cells were defined as having mean intensity >256 for KRT and >768 for nuclear Ki67. The difference of Gaussian algorithm implemented in scikit-image (v.0.19.3)^73^ was used to identify pRPA foci in segmented nuclei.

### Multiplexed immunohistochemistry

Tumor specimen slides were processed and stained as described^36^. ROIs across the primary tumor resections were selected based on tissue quality post-staining and annotated in Aperio ImageScope (Leica Biosystems). LA regions were selected based on visual identification of cell clusters containing >20 cells, positively stained with CD20 (B cell) and CD3 (T cell), within 500 µm. Data were processed as described^36,74^. In brief, images were registered using MATLAB (The MathWorks), AEC signal was extracted using Fiji^75^, single-cell segmentation and labeling was performed using StarDist 2D^76^, the mean signal intensity of each cell for every marker was measured using CellProfiler^77^ and gating thresholds were set using FCS Express Image Cytometry (De Novo Software). Cell-type gating and cell type counts are in source data.

### TCRβ sequencing and analysis

Frozen leukocytes and 25-mm thick curls of FFPE tumor were submitted to Adaptive Biotechnologies for human TCRβ sequencing. The tumor specimens were categorized as primary or metastasis. The blood specimens were also categorized as associated with primary disease or metastatic disease based on the following criteria. The blood sample was considered primary-associated if it was collected before or on the day of primary tumor resection, metastasis-associated if it was collected after a recurrence, or uncharacterized if it was collected after resection and before recurrence. For patients not treated by resection, the blood was considered primary-associated if it was collected 180 days before the latest date the patient was confirmed metastasis free on imaging. The blood was considered metastasis-associated if it was collected after the patient had metastasis confirmed on imaging or was collected within 30 days before metastasis was detected on imaging. Analyses were performed using the Immunoseq tool^78^ provided by Adaptive Biotechnologies and custom code (https://github.com/engjen/Liver_Lung_PDAC). Samples with fewer than 100 productive templates were excluded from analyses. The Diversity Metrics Tool was used for richness and evenness metrics, and the differential abundance tool was used to assess overlap between samples from 214 matched pairs of tumor and blood (91% collected on the same day). The percentage of tumor-distinct clones was calculated from a list of all rearrangements with ≥10 templates in each patient’s blood plus tumor samples combined, where tumor-distinct clones were defined as those found in tumor samples, but not found in matched blood samples. For shared, dominant clonal sequences within cohorts, the top 50 CDR3 rearrangement amino acid sequences (by frequency in each sample) were compiled for all samples, and the Immunoseq Sequence Search Tool was used to identify all samples in the cohort that contained any of those CDR3 TCRβ sequences at any frequency. Only the CDR3 amino acid sequences found in at least 25% of samples in the cohort were considered shared, dominant clonal sequences. Shannon entropy, clonality, Simpson’s *d*, tumor-distinct clone sequences, number of templates per sample, patient-level shared, dominant clonal sequences, expanded clones and putative KRAS-specific sequences were calculated from amino acid CDR3 frequency using scipy (v.1.11.4) and numpy (v.1.26.2). Python libraries and custom code are found at https://github.com/engjen/Liver_Lung_PDAC. Repertoire overlap was calculated using the repOverlap function from immunarch (v.0.9.0) in R and selecting ‘public’ and ‘Jaccard’ methods. To summarize the amount of shared TCRs per sample, we calculated each sample’s public overlap and Jaccard index with every other sample’s TCR repertoire and took the mean, indicative of a sample’s average T cell responses to common antigens.

### External datasets

The TCGA PAAD dataset was obtained from cbioportal^24^ and filtered for PDAC samples, resulting in *n* = 140. The ICGC PDAC RNA-seq specimen dataset was the APGI *n* = 96 specimen cohort (*n* = 87 with survival metadata)^9^, part of the ICGC study.

### Software

R (v.3.6.0) was used for GSVA and VIPER. R (v.4.1.2) was used with R packages DESeq2, GSVA, msigdbr, gplots and ggplot. R (v.4.2.2) was used with R packages Seurat, enrichplot and ClusterProfiler. GSEA was run in JAVA using the command line interface. Statistical tests were performed with R and Python (v.3.9.15). Environment information, data and code necessary to reproduce all paper figures are available at https://github.com/engjen/Liver_Lung_PDAC.

### Statistics and reproducibility

No statistical method was used to predetermine sample sizes, but our sample sizes for clinical and transcriptomic analysis compare favorably to those reported in previous publications (ICGC^9^, TCGA^24^ and COMPASS^79^). Liver (*n* = 9) and lung (*n* = 4) cohort samples included in the TMA were selected before transcriptomic analysis and based on tissue availability; sample size was constrained by available array space. All liver/lung cohort samples on the TMA were also profiled by mIHC, but we excluded one lung cohort sample from analysis due to quality control failure. No randomization was performed in our study as it is retrospective. Blinding was not used in any aspect of our study except during histological data appraisal by pathologists, who were blinded to study cohorts. A log-rank test was used to compare K–M survival and recurrence curves as indicated in figures. To determine optimal cutoffs for binarizing pORG, pSUB and PurIST GSVA scores and TCR metrics scores into high and low for survival analysis, we used the R package ROCit or sklearn.metrics.roc_curve to generate a receiver-operator curve comparing specificity and sensitivity of different cutoffs to predict short-term survivors (<545 days) versus long-term survivors (>545 days). We selected our optimal cutoff at the maximum Youden’s index (the value giving maximum sensitivity + specificity for short-term versus long-term survivor prediction). For GSVA scores, this cutoff was externally validated for prognostic significance in the ICGC PDAC and TGGA PAAD datasets. CPH modeling was used to estimate HRs for survival and recurrence with associated *P* values. For all survival analysis, only patients alive 30 days or more after surgery were included to avoid analyzing death related to surgical complications. Two-tailed *t*-tests were used when comparing two conditions and analysis of variance (ANOVA) was used when comparing more than two conditions within a dataset. Data normality was assessed using Q-Q plots. For non-Gaussian data (for example, Simpson’s evenness of TCR sequences and tumor-distinct clones) we used Kruskal–Wallis tests, or log transformed and applied ANOVA or two-tailed *t*-tests if data were log-normal (for example, TCR productive rearrangements, Simpson’s diversity, public clonotypes and Jaccard index). Pearson, Spearman and Kendall tau correlation coefficients were generated for Gaussian, non-Gaussian and censored data, respectively. Two-sided Fisher’s exact tests were used for 2 × 2 categorical comparisons and two-way chi-squared was used for categorical comparisons with more categories. McNemar’s tests were used for paired categorical data. FDR multiple comparisons correction was applied using the Benjamini–Hochberg method.

### Reporting summary

Further information on research design is available in the Nature Portfolio Reporting Summary linked to this article.

## Supplementary information

Reporting Summary

## Source data

Source Data Fig. 1 Statistical source data.

Source Data Fig. 2 Statistical source data.

Source Data Fig. 3 Statistical source data.

Source Data Fig. 4 Statistical source data.

Source Data Fig. 5 Statistical source data.

Source Data Fig. 6 Statistical source data.

Source Data Fig. 7 Statistical source data.

Source Data Extended Data Fig. 1 Statistical source data.

Source Data Extended Data Fig. 2 Statistical source data.

Source Data Extended Data Fig. 3 Statistical source data.

Source Data Extended Data Fig. 4 Statistical source data.

Source Data Extended Data Fig. 5 Statistical source data.

Source Data Extended Data Fig. 6 Statistical source data.

Source Data Extended Data Fig. 7 Statistical source data.

Source Data Extended Data Fig. 8 Statistical source data.

Source Data Extended Data Fig. 9 Statistical source data.

## Data Availability

All data generated for this study are available as follows: DNA sequencing and variant data from the xT gene panel, and the RNA-seq data are accessible through the NCI Genomic Data Commons deposited in the controlled access database dbGaP under accession phs003597.v1.p1. In accordance with informed patient consent for use and collection of these samples and generated data, use of this dataset is restricted to research pertaining to the study of pancreas disease. According to NIH policy, access through the data portal is limited to senior-level investigators (tenure-track professor, senior scientist or equivalent). Requests to access the genomic data must be submitted to dbGaP at https://dbgap.ncbi.nlm.nih.gov. The summarized, gene-level RNA-seq data are available in the GEO database under accession code GSE281129. TCR sequence data are available on the Adaptive Biotechnologies platform or in the GEO database under accession code GSE281129. The multiplexed immunofluorescence images, segmentation masks and extracted features are available at https://www.synapse.org/#!Synapse:syn51068458/wiki/620854. The mIHC single-cell phenotype and location data are available at https://www.synapse.org/#!Synapse:syn51078766. Source data for Figs. 1–7 and Extended Data Figs. 1–9 have been provided as Source Data files. The external datasets analyzed are available at https://static-content.springer.com/esm/art%3A10.1038%2Fnature16965/MediaObjects/41586_2016_BFnature16965_MOESM271_ESM.xlsx (ICGC), https://cbioportal-datahub.s3.amazonaws.com/paad_tcga_pan_can_atlas_2018.tar.gz and https://www.cbioportal.org/study/summary?id=paad_tcga_pan_can_atlas_2018 (TCGA). Human genome Release 24 (GRCh38.p5) is at https://www.gencodegenes.org/human/release_24.html. Source data are provided with this paper.

## Code availability

Free and open-source code and data used for analysis and all figures in this work are available at https://github.com/engjen/Liver_Lung_PDAC. An explanation of the repository source data is included in the data dictionary: Source Datasets README.txt.

## References

[1] Downs-Canner, S. et al. The indolent nature of pulmonary metastases from ductal adenocarcinoma of the pancreas. *J. Surg. Oncol.* **112**, 80–85 (2015). 26153355 10.1002/jso.23943 PMC4509861

[2] He, C., Huang, X., Zhang, Y., Lin, X. & Li, S. The impact of different metastatic patterns on survival in patients with pancreatic cancer. *Pancreatology* **21**, 556–563 (2021). 33518454 10.1016/j.pan.2021.01.014

[3] Wangjam, T. et al. Resected pancreatic ductal adenocarcinomas with recurrence limited in lung have a significantly better prognosis than those with other recurrence patterns. *Oncotarget* **6**, 36903–36910 (2015). 26372811 10.18632/oncotarget.5054 PMC4742219

[4] Link, J. M. et al. Tumor-infiltrating leukocyte phenotypes distinguish outcomes in related patients with pancreatic adenocarcinoma. *JCO Precis. Oncol.* **5**, 344–356 (2021). 10.1200/PO.20.00287 PMC8140804 34036232

[5] Arnaoutakis, G. J. et al. Pulmonary resection for isolated pancreatic adenocarcinoma metastasis: an analysis of outcomes and survival. *J. Gastrointest. Surg.* **15**, 1611–1617 (2011). 21725701 10.1007/s11605-011-1605-8 PMC3160502

[6] Houg, D. S. & Bijlsma, M. F. The hepatic pre-metastatic niche in pancreatic ductal adenocarcinoma. *Mol. Cancer* **17**, 95 (2018). 29903049 10.1186/s12943-018-0842-9 PMC6003100

[7] Lee, J. W. et al. Hepatocytes direct the formation of a pro-metastatic niche in the liver. *Nature* **567**, 249–252 (2019). 30842658 10.1038/s41586-019-1004-y PMC6430113

[8] Yu, J. et al. Liver metastasis restrains immunotherapy efficacy via macrophage-mediated T cell elimination. *Nat. Med.* **27**, 152–164 (2021). 33398162 10.1038/s41591-020-1131-x PMC8095049

[9] Bailey, P. et al. Genomic analyses identify molecular subtypes of pancreatic cancer. *Nature* **531**, 47–52 (2016). 26909576 10.1038/nature16965

[10] Chan-Seng-Yue, M. et al. Transcription phenotypes of pancreatic cancer are driven by genomic events during tumor evolution. *Nat. Genet.* **52**, 231–240 (2020). 31932696 10.1038/s41588-019-0566-9

[11] Collisson, E. A. et al. Subtypes of pancreatic ductal adenocarcinoma and their differing responses to therapy. *Nat. Med.* **17**, 500–503 (2011). 21460848 10.1038/nm.2344 PMC3755490

[12] Rashid, N. U. et al. Purity independent subtyping of tumors (PurIST), a clinically robust, single-sample classifier for tumor subtyping in pancreatic cancer. *Clin. Cancer Res.* **26**, 82–92 (2020). 31754050 10.1158/1078-0432.CCR-19-1467 PMC6942634

[13] Moffitt, R. A. et al. Virtual microdissection identifies distinct tumor- and stroma-specific subtypes of pancreatic ductal adenocarcinoma. *Nat. Genet.* **47**, 1168–1178 (2015). 26343385 10.1038/ng.3398 PMC4912058

[14] Puleo, F. et al. Stratification of pancreatic ductal adenocarcinomas based on tumor and microenvironment features. *Gastroenterology* **155**, 1999–2013.e1993 (2018). 30165049 10.1053/j.gastro.2018.08.033

[15] Halbrook, C. J., Lyssiotis, C. A., Pasca di Magliano, M. & Maitra, A. Pancreatic cancer: advances and challenges. *Cell* **186**, 1729–1754 (2023). 37059070 10.1016/j.cell.2023.02.014 PMC10182830

[16] Dreyer, S. B. et al. Targeting DNA damage response and replication stress in pancreatic cancer. *Gastroenterology* **160**, 362–377.e313 (2021). 33039466 10.1053/j.gastro.2020.09.043 PMC8167930

[17] Labrie, M., Brugge, J. S., Mills, G. B. & Zervantonakis, I. K. Therapy resistance: opportunities created by adaptive responses to targeted therapies in cancer. *Nat. Rev. Cancer* **22**, 323–339 (2022). 35264777 10.1038/s41568-022-00454-5 PMC9149051

[18] Morrison, A. H., Byrne, K. T. & Vonderheide, R. H. Immunotherapy and prevention of pancreatic cancer. *Trends Cancer* **4**, 418–428 (2018). 29860986 10.1016/j.trecan.2018.04.001 PMC6028935

[19] Zheng, L. et al. Vaccine-induced intratumoral lymphoid aggregates correlate with survival following treatment with a neoadjuvant and adjuvant vaccine in patients with resectable pancreatic adenocarcinoma. *Clin. Cancer Res.* **27**, 1278–1286 (2021). 33277370 10.1158/1078-0432.CCR-20-2974 PMC7925374

[20] Balachandran, V. P. et al. Identification of unique neoantigen qualities in long-term survivors of pancreatic cancer. *Nature* **551**, 512–516 (2017). 29132146 10.1038/nature24462 PMC6145146

[21] Pishvaian, M. J. et al. Overall survival in patients with pancreatic cancer receiving matched therapies following molecular profiling: a retrospective analysis of the Know Your Tumor registry trial. *Lancet Oncol.* **21**, 508–518 (2020). 32135080 10.1016/S1470-2045(20)30074-7 PMC7453743

[22] Love, M. I., Huber, W. & Anders, S. Moderated estimation of fold change and dispersion for RNA-seq data with DESeq2. *Genome Biol*. **15**, 550 (2014). 25516281 10.1186/s13059-014-0550-8 PMC4302049

[23] Hanzelmann, S., Castelo, R. & Guinney, J. GSVA: gene set variation analysis for microarray and RNA-seq data. *BMC Bioinform.* **14**, 7 (2013). 10.1186/1471-2105-14-7 PMC3618321 23323831

[24] Cancer Genome Atlas Research Network. Integrated genomic characterization of pancreatic ductal adenocarcinoma. *Cancer Cell* 32, 185–203.e113 (2017). 10.1016/j.ccell.2017.07.007 PMC5964983 28810144

[25] Werba, G. et al. Single-cell RNA sequencing reveals the effects of chemotherapy on human pancreatic adenocarcinoma and its tumor microenvironment. *Nat. Commun.* **14**, 797 (2023). 36781852 10.1038/s41467-023-36296-4 PMC9925748

[26] Liberzon, A. et al. The Molecular Signatures Database (MSigDB) hallmark gene set collection. *Cell Syst*. **1**, 417–425 (2015). 26771021 10.1016/j.cels.2015.12.004 PMC4707969

[27] Alvarez, M. J. et al. Functional characterization of somatic mutations in cancer using network-based inference of protein activity. *Nat. Genet.* **48**, 838–847 (2016). 27322546 10.1038/ng.3593 PMC5040167

[28] Lachmann, A., Giorgi, F. M., Lopez, G. & Califano, A. ARACNe-AP: gene network reverse engineering through adaptive partitioning inference of mutual information. *Bioinformatics* **32**, 2233–2235 (2016). 27153652 10.1093/bioinformatics/btw216 PMC4937200

[29] Heeke, A. L. et al. Prevalence of homologous recombination-related gene mutations across multiple cancer types. *JCO Precis. Oncol.* **2**, 1–13 (2018). 10.1200/PO.17.00286 PMC6139373 30234181

[30] Duarte, C. W. et al. Expression signature of IFN/STAT1 signaling genes predicts poor survival outcome in glioblastoma multiforme in a subtype-specific manner. *PLoS ONE* **7**, e29653 (2012). 22242177 10.1371/journal.pone.0029653 PMC3252343

[31] Khodarev, N. N. et al. STAT1 is overexpressed in tumors selected for radioresistance and confers protection from radiation in transduced sensitive cells. *Proc. Natl Acad. Sci. USA* **101**, 1714–1719 (2004). 14755057 10.1073/pnas.0308102100 PMC341831

[32] Weichselbaum, R. R. et al. An interferon-related gene signature for DNA damage resistance is a predictive marker for chemotherapy and radiation for breast cancer. *Proc. Natl Acad. Sci. USA* **105**, 18490–18495 (2008). 19001271 10.1073/pnas.0809242105 PMC2587578

[33] Musella, M., Galassi, C., Manduca, N. & Sistigu, A. The yin and yang of type I IFNs in cancer promotion and immune activation. *Biology* **10**, 856 (2021). 34571733 10.3390/biology10090856 PMC8467547

[34] Boukhaled, G. M., Harding, S. & Brooks, D. G. Opposing roles of type I interferons in cancer immunity. *Annu. Rev. Pathol.* **16**, 167–198 (2021). 33264572 10.1146/annurev-pathol-031920-093932 PMC8063563

[35] Banik, G. et al. High-dimensional multiplexed immunohistochemical characterization of immune contexture in human cancers. *Methods Enzymol.* **635**, 1–20 (2020). 32122539 10.1016/bs.mie.2019.05.039 PMC7390987

[36] Liudahl, S. M. et al. Leukocyte heterogeneity in pancreatic ductal adenocarcinoma: phenotypic and spatial features associated with clinical outcome. *Cancer Discov*. **11**, 2014–2031 (2021). 33727309 10.1158/2159-8290.CD-20-0841 PMC8338775

[37] Arunkumar, M. & Zielinski, C. E. T-cell receptor repertoire analysis with computational tools-an immunologist’s perspective. *Cells* **10**, 3582 (2021). 34944090 10.3390/cells10123582 PMC8700004

[38] Chiffelle, J. et al. T-cell repertoire analysis and metrics of diversity and clonality. *Curr. Opin. Biotechnol.* **65**, 284–295 (2020). 32889231 10.1016/j.copbio.2020.07.010

[39] Hopkins, A. C. et al. T cell receptor repertoire features associated with survival in immunotherapy-treated pancreatic ductal adenocarcinoma. *JCI Insight* **3**, e122092 (2018). 29997287 10.1172/jci.insight.122092 PMC6124515

[40] Li, N., Yuan, J., Tian, W., Meng, L. & Liu, Y. T-cell receptor repertoire analysis for the diagnosis and treatment of solid tumor: a methodology and clinical applications. *Cancer Commun.* **40**, 473–483 (2020). 10.1002/cac2.12074 PMC7571402 32677768

[41] Sims, J. S. et al. Diversity and divergence of the glioma-infiltrating T-cell receptor repertoire. *Proc. Natl Acad. Sci. USA* **113**, E3529–E3537 (2016). 27261081 10.1073/pnas.1601012113 PMC4922177

[42] Schumacher, T. N. & Thommen, D. S. Tertiary lymphoid structures in cancer. *Science* **375**, eabf9419 (2022). 34990248 10.1126/science.abf9419

[43] Lu, D. et al. KRAS G12V neoantigen specific T cell receptor for adoptive T cell therapy against tumors. *Nat. Commun.* **14**, 6389 (2023). 37828002 10.1038/s41467-023-42010-1 PMC10570350

[44] Tran, E. et al. T-cell transfer therapy targeting mutant KRAS in cancer. *N. Engl. J. Med.* **375**, 2255–2262 (2016). 27959684 10.1056/NEJMoa1609279 PMC5178827

[45] Lowery, F. J. et al. Molecular signatures of antitumor neoantigen-reactive T cells from metastatic human cancers. *Science* **375**, 877–884 (2022). 35113651 10.1126/science.abl5447 PMC8996692

[46] Bear, A. S. et al. Biochemical and functional characterization of mutant KRAS epitopes validates this oncoprotein for immunological targeting. *Nat. Commun.* **12**, 4365 (2021). 34272369 10.1038/s41467-021-24562-2 PMC8285372

[47] Knudsen, E. S. et al. Stratification of pancreatic ductal adenocarcinoma: combinatorial genetic, stromal, and immunologic markers. *Clin. Cancer Res.* **23**, 4429–4440 (2017). 28348045 10.1158/1078-0432.CCR-17-0162 PMC5951386

[48] Daemen, A. et al. Metabolite profiling stratifies pancreatic ductal adenocarcinomas into subtypes with distinct sensitivities to metabolic inhibitors. *Proc. Natl Acad. Sci. USA* **112**, E4410–E4417 (2015). 26216984 10.1073/pnas.1501605112 PMC4538616

[49] Beaubier, N. et al. Integrated genomic profiling expands clinical options for patients with cancer. *Nat. Biotechnol.* **37**, 1351–1360 (2019). 31570899 10.1038/s41587-019-0259-z

[50] Beaubier, N. et al. Clinical validation of the tempus xT next-generation targeted oncology sequencing assay. *Oncotarget* **10**, 2384–2396 (2019). 31040929 10.18632/oncotarget.26797 PMC6481324

[51] Gu, Z., Eils, R. & Schlesner, M. Complex heatmaps reveal patterns and correlations in multidimensional genomic data. *Bioinformatics* **32**, 2847–2849 (2016). 27207943 10.1093/bioinformatics/btw313

[52] Bray, N. L., Pimentel, H., Melsted, P. & Pachter, L. Near-optimal probabilistic RNA-seq quantification. *Nat. Biotechnol.* **34**, 525–527 (2016). 27043002 10.1038/nbt.3519

[53] Koster, J. & Rahmann, S. Snakemake–a scalable bioinformatics workflow engine. *Bioinformatics* **28**, 2520–2522 (2012). 22908215 10.1093/bioinformatics/bts480

[54] Robinson, M. D., McCarthy, D. J. & Smyth, G. K. edgeR: a Bioconductor package for differential expression analysis of digital gene expression data. *Bioinformatics* **26**, 139–140 (2010). 19910308 10.1093/bioinformatics/btp616 PMC2796818

[55] Subramanian, A. et al. Gene set enrichment analysis: a knowledge-based approach for interpreting genome-wide expression profiles. *Proc. Natl Acad. Sci. USA* **102**, 15545–15550 (2005). 16199517 10.1073/pnas.0506580102 PMC1239896

[56] Nirmal, A. J. et al. Immune cell gene signatures for profiling the microenvironment of solid tumors. *Cancer Immunol. Res.* **6**, 1388–1400 (2018). 30266715 10.1158/2326-6066.CIR-18-0342

[57] Kolde, R. pheatmap: pretty heatmaps. R package version 1.0.12 (2019).

[58] Hao, Y. et al. Integrated analysis of multimodal single-cell data. *Cell* **184**, 3573–3587.e3529 (2021). 34062119 10.1016/j.cell.2021.04.048 PMC8238499

[59] Hafemeister, C. & Satija, R. Normalization and variance stabilization of single-cell RNA-seq data using regularized negative binomial regression. *Genome Biol.* **20**, 296 (2019). 31870423 10.1186/s13059-019-1874-1 PMC6927181

[60] Sturm, G. et al. Comprehensive evaluation of transcriptome-based cell-type quantification methods for immuno-oncology. *Bioinformatics* **35**, i436–i445 (2019). 31510660 10.1093/bioinformatics/btz363 PMC6612828

[61] Finotello, F. et al. Molecular and pharmacological modulators of the tumor immune contexture revealed by deconvolution of RNA-seq data. *Genome Med.* **11**, 34 (2019). 31126321 10.1186/s13073-019-0638-6 PMC6534875

[62] Becht, E. et al. Estimating the population abundance of tissue-infiltrating immune and stromal cell populations using gene expression. *Genome Biol*. **17**, 218 (2016). 27908289 10.1186/s13059-016-1113-y PMC5134277

[63] Aran, D., Hu, Z. & Butte, A. J. xCell: digitally portraying the tissue cellular heterogeneity landscape. *Genome Biol*. **18**, 220 (2017). 29141660 10.1186/s13059-017-1349-1 PMC5688663

[64] Racle, J., de Jonge, K., Baumgaertner, P., Speiser, D. E. & Gfeller, D. Simultaneous enumeration of cancer and immune cell types from bulk tumor gene expression data. *eLife* **6**, e26476 (2017). 29130882 10.7554/eLife.26476 PMC5718706

[65] Ashburner, M. et al. Gene Ontology: tool for the unification of biology. The Gene Ontology Consortium. *Nat. Genet.* **25**, 25–29 (2000). 10802651 10.1038/75556 PMC3037419

[66] The Gene Ontology Consortium et al. The Gene Ontology knowledgebase in 2023. *Genetics* **224**, iyad031 (2023). 10.1093/genetics/iyad031 PMC10158837 36866529

[67] Wu, T. et al. clusterProfiler 4.0: A universal enrichment tool for interpreting omics data. *Innovation* **2**, 100141 (2021). 34557778 10.1016/j.xinn.2021.100141 PMC8454663

[68] Yu, G., Hu, E. & Gao, C. H. enrichplot: visualization of functional enrichment result. R package version 1.18.4 (2023).

[69] Eng, J. et al. A framework for multiplex imaging optimization and reproducible analysis. *Commun. Biol.* **5**, 438 (2022). 35545666 10.1038/s42003-022-03368-y PMC9095647

[70] Stringer, C., Wang, T., Michaelos, M. & Pachitariu, M. Cellpose: a generalist algorithm for cellular segmentation. *Nat. Methods* **18**, 100–106 (2021). 33318659 10.1038/s41592-020-01018-x

[71] Greenwald, N. F. et al. Whole-cell segmentation of tissue images with human-level performance using large-scale data annotation and deep learning. *Nat. Biotechnol.* **40**, 555–565 (2022). 34795433 10.1038/s41587-021-01094-0 PMC9010346

[72] Wolf, F. A., Angerer, P. & Theis, F. J. SCANPY: large-scale single-cell gene expression data analysis. *Genome Biol*. **19**, 15 (2018). 29409532 10.1186/s13059-017-1382-0 PMC5802054

[73] van der Walt, S. et al. scikit-image: image processing in Python. *PeerJ* **2**, e453 (2014). 25024921 10.7717/peerj.453 PMC4081273

[74] Mi, H. et al. Quantitative spatial profiling of immune populations in pancreatic ductal adenocarcinoma reveals tumor microenvironment heterogeneity and prognostic biomarkers. *Cancer Res.* **82**, 4359–4372 (2022). 36112643 10.1158/0008-5472.CAN-22-1190 PMC9716253

[75] Schindelin, J. et al. Fiji: an open-source platform for biological-image analysis. *Nat. Methods* **9**, 676–682 (2012). 22743772 10.1038/nmeth.2019 PMC3855844

[76] Schmidt U, W. M. et al. in *Medical Image Computing and Computer Assisted Intervention – MICCAI* (eds Schnabel, J. A. et al.) 265–273 (Springer International Publishing, 2018).

[77] Carpenter, A. E. et al. CellProfiler: image analysis software for identifying and quantifying cell phenotypes. *Genome Biol.* **7**, R100 (2006). 17076895 10.1186/gb-2006-7-10-r100 PMC1794559

[78] Robins, H. S. et al. Comprehensive assessment of T-cell receptor β-chain diversity in αβ T cells. *Blood* **114**, 4099–4107 (2009). 19706884 10.1182/blood-2009-04-217604 PMC2774550

[79] Aung, K. L. et al. Genomics-driven precision medicine for advanced pancreatic cancer: early results from the COMPASS Trial. *Clin. Cancer Res.* **24**, 1344–1354 (2018). 29288237 10.1158/1078-0432.CCR-17-2994 PMC5968824

