# Supplementary material for: Ongoing replication stress tolerance and clonal T cell responses distinguish liver and lung recurrence and outcomes in pancreatic cancer
Source: Nat Cancer. 2025 Jan 9;6(1):123–44. doi: 10.1038/s43018-024-00881-3 (PMC11779630; doi:10.1038/s43018-024-00881-3)
Supplement: Supplementary file 1 — Reporting Summary [file 43018_2024_881_MOESM1_ESM.pdf]

nature portfolio

Corresponding author(s): Rosalie Sears

Last updated by author(s): Aug 26, 2024

Reporting Summary

Nature Portfolio wishes to improve the reproducibility of the work that we publish. This form provides structure for consistency and transparency in reporting. For further information on Nature Portfolio policies, see our Editorial Policies and the Editorial Policy Checklist.

Statistics

For all statistical analyses, confirm that the following items are present in the figure legend, table legend, main text, or Methods section.

n/a	Confirmed	
☐	☒	The exact sample size (*n*) for each experimental group/condition, given as a discrete number and unit of measurement
☐	☒	A statement on whether measurements were taken from distinct samples or whether the same sample was measured repeatedly
☐	☒	The statistical test(s) used AND whether they are one- or two-sided<br>*Only common tests should be described solely by name; describe more complex techniques in the Methods section.*
☐	☒	A description of all covariates tested
☐	☒	A description of any assumptions or corrections, such as tests of normality and adjustment for multiple comparisons
☐	☒	A full description of the statistical parameters including central tendency (e.g. means) or other basic estimates (e.g. regression coefficient) AND variation (e.g. standard deviation) or associated estimates of uncertainty (e.g. confidence intervals)
☐	☒	For null hypothesis testing, the test statistic (e.g. *F*, *t*, *r*) with confidence intervals, effect sizes, degrees of freedom and *P* value noted<br>*Give P values as exact values whenever suitable.*
☒	☐	For Bayesian analysis, information on the choice of priors and Markov chain Monte Carlo settings
☒	☐	For hierarchical and complex designs, identification of the appropriate level for tests and full reporting of outcomes
☐	☒	Estimates of effect sizes (e.g. Cohen's *d*, Pearson's *r*), indicating how they were calculated

Our web collection on statistics for biologists contains articles on many of the points above.

Software and code

Policy information about availability of computer code

Data collection

Image data for multiplexed immunofluorescence was collected on a Zeiss Axioscan Z1 using the Zeiss Zen Blue software v2.3. Image data for multiplexed immunohistochemistry was collected on a Leica Aperio AT2 scanner.

Data analysis

Code used for data analysis and all figures in this work is available at: https://github.com/engjen/Liver_Lung_PDAC.

Statistical, machine learning, image analysis, and graphing software used:
R versions v3.6.0, v4.1.2, and v4.2.2
python v3.9.15

RNA-Seq alignment and gene expression summaries:
kallisto v0.44.0
Bioconda package bioconductor-tximport v1.12.1
FastQC v0.11.8 and MultiQC v1.7
trim-galore v0.6.3
anaconda package management system (conda v4.8.2)
genome assembly GRCh38.p5 with gencode v24 annotation

Analysis of RNA-Seq Data:
PurIST subtype scores were calculated using software from: https://github.com/naimurashid/PurIST.
R packages - DESeq2 v1.42.1, edgeR v4.0.16, fdrtool v1.2.17

scRNA-Seq (single cell RNA-Seq) analysis:
R package Seurat v4.3.0

Pathway analysis:
GSEA v4.1.0 was run using the command line interface
R packages - GSEABase v1.64.0, GSVA v1.32.0, msigdbr v7.5.1, msigdbr v, org.Hs.eg.db v3.17.0

Graphing:
R packages - Cairo v1.6.2, ggplot2 v3.5.1, ggfortify v0.4.17, pheatmap v1.0.12, ComplexHeatmap v2.18.0, enrichplot v1.18.4, ClusterProfiler v4.6.2

Image analysis:
Matlab v9.11.0 (https://www.mathworks.com/products/matlab.html)
sklearn v1.0.2
scanpy v1.9.3 (https://github.com/theislab/Scanpy)
scikit-image v0.19.3 (http://scikit-image.org)
Cellpose [PMID: 33318659]
Mesmer [PMID: 34795433]
FIJI (https://doi.org/10.1038/nmeth.2019)
StarDist 2D (Schmidt U, W. M., Broaddus C, et al. . in Medical Image Computing and Computer Assisted Intervention – MICCAI (ed Schnabel JA Frangi AF, Davatzikos C, et al.) 265–273 (Springer International Publishing, 2018).)
FCS Express Image Cytometry (De Novo Software, Glendale, CA)

Survival analysis:
R packages - survival v3.6.4, ROCit v2.1.2

VIPER regulon enrichment analysis and Immune cell type estimation:
VIPER scores were calculated using the TCGA PAAD ARACNe-inferred network.
R packages - ClusterProfiler v4.6.2, immunedeconv v2.1.039 using algorithms: quantiseq4, mcp_counter, xcell, and epic.

Other:
R packages - XLConnect v1.0.10, enrichplot v1.22.0

For manuscripts utilizing custom algorithms or software that are central to the research but not yet described in published literature, software must be made available to editors and reviewers. We strongly encourage code deposition in a community repository (e.g. GitHub). See the Nature Portfolio guidelines for submitting code & software for further information.

Data

Policy information about availability of data

All manuscripts must include a data availability statement. This statement should provide the following information, where applicable:

- Accession codes, unique identifiers, or web links for publicly available datasets
- A description of any restrictions on data availability
- For clinical datasets or third party data, please ensure that the statement adheres to our policy

All data generated for this study are available as follows: DNA sequencing and variant data from the xT gene panel and the RNA-seq sequencing data are accessible through the NCI Genomic Data Commons deposited in the controlled access database dbGaP under accession phs003597.v1.p1: http://www.ncbi.nlm.nih.gov/projects/gap/cgi-bin/study.cgi?study_id=phs003597.v1.p1. In accordance with informed patient consent for use and collection of these samples and generated data, use of this dataset is restricted to research pertaining to the study of pancreas disease. According to NIH policy, access through the data portal is limited to senior level investigators (tenure-track professor, senior scientist, or equivalent). Requests to access the genomic data must be submitted to dbGaP at https://dbgap.ncbi.nlm.nih.gov. The summarized, gene level RNA-seq data is available in the Gene Expression Omnibus (GEO) database under accession GSE281129: https://www.ncbi.nlm.nih.gov/geo/query/acc.cgi?acc=GSE281129. TCR sequence data is available on the Adaptive Biotechnologies platform, or in the Gene Expression Omnibus (GEO) database under accession GSE281129. The multiplexed immunofluorescence images, segmentation masks and extracted features are available at: https://www.synapse.org/#!Synapse:syn51068458/wiki/620854. The multiplexed immunohistochemistry single cell phenotype and location data are available: https://www.synapse.org/#!Synapse:syn51078766. Source data for Fig. 1-7 and Extended Data Fig. 1-9 have been provided as Source Data files. External datasets analyzed are available at https://static-content.springer.com/esm/art%3A10.1038%2Fnature16965/MediaObjects/41586_2016_BFnature16965_MOESM271_ESM.xlsx (ICGC) and https://cbioportal-datahub.s3.amazonaws.com/paad_tcga_pan_can_atlas_2018.tar.gz and https://www.cbioportal.org/study/summary?id=paad_tcga_pan_can_atlas_2018 (TCGA). Human genome Release 24 (GRCh38.p5): https://www.gencodegenes.org/human/release_24.html.

Research involving human participants, their data, or biological material

Policy information about studies with human participants or human data. See also policy information about sex, gender (identity/presentation), and sexual orientation and race, ethnicity and racism.

Reporting on sex and gender	Neither sex nor gender was used to select patients or specimens used in this study. Sex was self-reported and obtained from the medical records. The numbers of patients by sex are given in Table 1 and as disaggregated data is in Source Dataset 1. Patients in the study consent to individual demographic data sharing. There are 193 females and 229 males in the study. Sex was considered as a variable in the Cox proportional hazards survival analysis (Source dataset 2) and tested for sex differences across the study cohorts in Table 1.
Reporting on race, ethnicity, or	We included self reported race in Table 1. We did not report on ethnicity. The participants in our study only included White,

Reporting on race, ethnicity, or other socially relevant	Asian and Unknown based on self-reporting in the electronic medical records. Race was tested and found to not be a confounding variable in our study, see Table 1.
Population characteristics	Demographic and clinical covariates known are given in Table 1 and Source Dataset 1, including age, treatment, stage, grade and primary tumor site.
Recruitment	All patients treated for pancreatic adenocarcinoma at Oregon Health & Science University are given the option to consent to the Oregon Pancreatic Tissue Registry. Only specimens from consented patients were used for this study. Our center is a referral site for the whole state of Oregon and the majority of our patients agree to consent which provides us with a representative sample of patients. Many patients with advanced disease do not qualify for surgery which biases our collection of primary tumors for RNA-Seq and DNA gene panel analysis.
Ethics oversight	Our research complies with all relevant ethical regulations and was approved under Oregon Health & Science University (OHSU) IRB protocol #00003609. Patient data, blood, and tissues were obtained with informed consent in accordance with the Declaration of Helsinki and were acquired through the Oregon Pancreas Tissue Registry. Patients were not compensated for participation.

Note that full information on the approval of the study protocol must also be provided in the manuscript.

Field-specific reporting

Please select the one below that is the best fit for your research. If you are not sure, read the appropriate sections before making your selection.

☒ Life sciences ☐ Behavioural & social sciences ☐ Ecological, evolutionary & environmental sciences

For a reference copy of the document with all sections, see nature.com/documents/nr-reporting-summary-flat.pdf

Life sciences study design

All studies must disclose on these points even when the disclosure is negative.

Sample size	Sample sizes are given for each result on figures or in figure legends. Sample size was constrained by available samples in our registry over a 5 year period prior to our study that allowed for at least 2 years of follow up. We did not perform a power analysis, but our sample size compares favorably with similar, public data sets (e.g., TCGA PAAD and ICGC APGI). In most cases, our sample numbers were in the hundreds and more than sufficient. In some cases, when comparing cohorts, some cohorts had limited numbers (e.g., basal-like cohorts) which did limit the statistical significance of results as noted in the manuscript).
Data exclusions	Data exclusions are given in figure legends. Patients without pancreatic adenocarcinoma were excluded. Patients who died within 30 days of primary tumor surgical resection were excluded from survival outcomes analyses.
Replication	Where possible, analyses of data from OHSU were also applied to publicly available datasets (e.g., TCGA PAAD and ICGC APGI). We were able to replicate our main findings regarding pORG and pSUB scores and survival in both TCGA PAAD and ICGC APGI datasets. We did not have access to independent datasets with primary PDAC with metastatic site information. To address this, we performed leave-one-out (LOO) cross validation within our dataset and reported in methods under pORG gene set generation that LOO cross validation significantly called liver or lung cohort primary tumors in the left-out samples. We also changed the language regarding pORG and metastatic tropism to an "association" and we stated that independent datasets with known metastatic site information are needed to replicate our study.
Randomization	For some comparisons, tumor specimens were assigned scores and patients were assigned to cohorts. The scores and cohort assignments were made while investigators were blinded.
Blinding	Investigators were blinded to tumor and blood specimen type and origin when performing analyses and scoring. Clinical data were collected before the study and static throughout the study.

Reporting for specific materials, systems and methods

We require information from authors about some types of materials, experimental systems and methods used in many studies. Here, indicate whether each material, system or method listed is relevant to your study. If you are not sure if a list item applies to your research, read the appropriate section before selecting a response.

Materials & experimental systems

n/a	Involved in the study
☒	☐ Antibodies
☒	☐ Eukaryotic cell lines
☒	☐ Palaeontology and archaeology
☒	☐ Animals and other organisms
☒	☐ Clinical data
☒	☐ Dual use research of concern
☒	☐ Plants

Methods

n/a	Involved in the study
☒	☐ ChIP-seq
☒	☐ Flow cytometry
☒	☐ MRI-based neuroimaging

Plants

Seed stocks	n/a
Novel plant genotypes	n/a
Authentication	n/a
